# Tortoise Oligopeptides Augment Cyclophosphamide's Antitumor Activity Through Dual Modulation of Therapeutic Efficacy and Hematologic Toxicity

**DOI:** 10.1002/fsn3.71078

**Published:** 2025-10-16

**Authors:** Zhuotao Fu, Tian Yu, Cong Meng, Guoni Zhang, Shaojun Huang, Yuping Li, Linchun Fu, Zhitong Deng

**Affiliations:** ^1^ The First Affiliated Hospital of Guangzhou University of Chinese Medicine Guangzhou China; ^2^ Guangdong Clinical Research Academy of Chinese Medicine Guangzhou China; ^3^ Science and Technology Innovation Center Guangzhou University of Chinese Medicine Guangzhou China; ^4^ The Affiliated TCM Hospital of Guangzhou Medical University Guangzhou China; ^5^ Haikou Hospital of Traditional Chinese Medicine Haikou China

**Keywords:** diet therapy, gut microbiota, leukopenia, small molecule peptide, tumor, ultrafiltration molecular weight peptides

## Abstract

Cyclophosphamide (CTX) is a widely used chemotherapeutic agent, but its efficacy is often limited by leukopenia, a common adverse effect for which effective preventive strategies are currently lacking. In this study, oligopeptides were prepared from a blend of three edible tortoise species ( *Cuora trifasciata* , *Mauremys mutica* , and *Chinemys reevesii* ) and evaluated for their potential to alleviate CTX‐induced leukopenia. The resulting Tortoise Oligopeptides (TOPs) were characterized primarily as small molecules with molecular weights under 5 kDa and peptide lengths between 4 and 15 amino acids, rich in glycine, glutamic acid, and proline. In a mouse model, TOPs administration significantly ameliorated CTX‐induced leukopenia in a dose‐dependent manner, attenuated pathological damage in the spleen and femur, and correlated with elevated serum levels of IL‐4, IL‐1β, TNF‐α, and IFN‐γ. In CTX‐treated tumor‐bearing mice, TOPs not only reduced leukopenia but also enhanced the antitumor efficacy of CTX. Correlation analyses linked leukocyte recovery to increased relative abundance of gut microbiota genera such as *Colidextribacter*, *Tyzzerella*, *Prevotellaceae_UCG_001*, and *Rikenella*. KEGG pathway analysis and fecal microbiota transplantation (FMT) experiments indicated that TOPs alleviate CTX‐induced granulocytopenia partly through modulation of the gut microbiota. Additionally, LC–MS/MS sequencing combined with bioinformatic prediction and molecular docking identified several peptides—including PAIPAPPVGPGPK, FSFPTLPF, and PGLPFHP—with high binding affinity to key tumor targets (BCL‐2, MDM2, EGFR), suggesting intrinsic antitumor properties. These findings indicate that TOPs may serve as a specialized medical food to mitigate CTX‐induced leukopenia through multimodal mechanisms involving immunonutrition, gut microbiota regulation, and direct antitumor peptide effects.

## Graphical abstract

Schematic summary of the synergistic antitumor and myeloprotective effects of TOPs in CTX‐treated tumor‐bearing mice, mediated through gut microbiota modulation and direct peptide‐target interactions. CTX, Cyclophosphamide; FMT, Fecal Microbiota Transplantation; S180, S180 tumor cells; TOPs, Tortoise Oligopeptides.

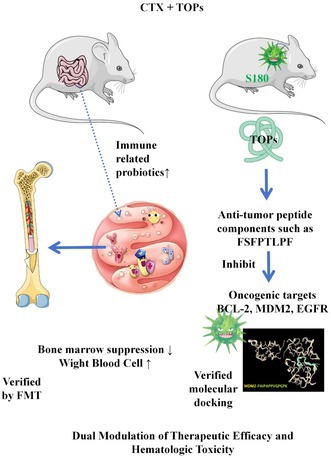

**Abbreviations**
ANOVA: analysis of variance
BCL‐2: B‐cell Lymphoma 2
CTX: cyclophosphamide
DTT: dithiothreitol
EGFR: epidermal growth factor receptor
ELISA: enzyme‐linked immunosorbent assay
FMT: fecal microbiota transplantation
HD: high‐dose group
H&E: hematoxylin and eosin
IAA: iodoacetamide
IFN‐γ: interferon‐gamma
IL‐1β: interleukin‐1 beta
IL‐4: interleukin‐4
kDa: Kilodalton
KEGG: kyoto encyclopedia of genes and genomes
KM mice: kunming mice
LC–MS/MS: liquid chromatography–tandem mass spectrometry
LD: low‐dose group
MC: model control group
MD: medium‐dose group
MDM2: mouse double Minute 2 homolog
NC: normal control group
PDB: Protein Data Bank
rhG‐CSF: recombinant human granulocyte colony‐stimulating factor
SCFAs: short‐chain fatty acids
s.e.m: standard error of the mean
SI: Spleen Index
SPF: specific pathogen‐free
TCTX: tumor‐bearing CTX group
TCTX‐R: tumor‐bearing CTX recipient group
THD: tumor‐bearing high‐dose group
THD‐R: tumor‐bearing high‐dose recipient group
TI: Thymus Index
TMC: tumor‐bearing model control group
TNF‐α: tumor necrosis factor‐alpha
TOPs: tortoise oligopeptides
WBC: white blood cell

## 1 Introduction

Cancer remains one of the most prevalent diseases worldwide, and Cyclophosphamide (CTX) is widely employed as a first‐line chemotherapeutic agent for various malignancies (Emadi et al. 2009). However, its clinical utility is often limited by severe adverse effects, particularly leukopenia resulting from bone marrow suppression (Zhou et al. 2023). Currently, recombinant human granulocyte colony‐stimulating factor (rhG‐CSF) represents the primary treatment for chemotherapy‐induced leukopenia. Although rhG‐CSF effectively promotes neutrophil production and function, emerging evidence suggests it may also inadvertently stimulate tumor growth, raising concerns regarding its safety profile in oncological settings (Sun et al. 2018; Wang et al. 2022). Thus, the development of strategies that mitigate CTX‐induced hematologic toxicity without compromising its antitumor efficacy—while simultaneously supporting immune recovery—constitutes a significant clinical challenge.

Additionally, malnutrition and gastrointestinal dysfunction are common among cancer patients undergoing chemotherapy, underscoring the need for easily absorbable nutritional supports that can help maintain physiological function and treatment tolerance.

Tortoise meat has a longstanding history in traditional Chinese medicine due to its purported immune‐enhancing properties. It has been consumed in various forms throughout China, notably in the well‐known tortoise jelly (Guilinggao) (Yip et al. 2005), and is described in classical texts such as *The Chinese Materia Medica* and *Huangdi Neijing* (*The Yellow Emperor's Inner Canon*) as nourishing the kidney meridian—a concept traditionally linked to bone marrow production and hematopoiesis. Modern science confirms that leukocytes originate in the bone marrow, suggesting a potential mechanistic connection between tortoise‐derived compounds and hematopoietic recovery. Furthermore, small oligopeptides exhibit superior absorbability compared to intact proteins, rendering them promising candidates for nutritional support in debilitated patients (Nielsen et al. 2017).

Based on this rationale, we hypothesized that oligopeptides derived from tortoise meat could alleviate CTX‐induced leukopenia. Three species— *Cuora trifasciata* , *Mauremys mutica* , and *Chinemys reevesii* —are commonly farmed in China for consumption (Zhao 2018) and have been preliminarily associated with anti‐tumor and immunomodulatory activities in vitro (He 2015; Lv et al. 2019; Shi et al. 2018). Nevertheless, evidence from in vivo models remains scarce.

In this study, we utilized a blended oligopeptide preparation derived from the meat of these three tortoise species in a 1:1:1 ratio. This formulation strategy was adopted for two principal reasons: firstly, it mirrors the composition of traditional tortoise jelly and other commercially available health products wherein multiple tortoise species are commonly used in combination (Lei 2025), thereby enhancing the translational relevance of our findings to existing products; secondly, from a practical standpoint, blending materials from multiple sources helps mitigate batch‐to‐batch variability inherent to single‐species use, ensuring a more consistent and representative profile of bioactive peptides derived from farmed tortoises as a food category. While this approach does not permit attribution of efficacy to any single species—a focus for subsequent research—it enables a holistic evaluation of their combined effects.

Accordingly, we prepared and characterized oligopeptides from this blended source and investigated their effects on leukopenia, immune organ pathology, gut microbiota, and antitumor efficacy in CTX‐treated mouse models. Our work aims to provide experimental evidence supporting the potential application of Tortoise Oligopeptides (TOPs) as a specialized medical food to ameliorate chemotherapy‐induced side effects and enhance treatment outcomes.

## 2 Experimental Design

### 2.1 Drugs and Reagents

CTX (Endoxan) was obtained from AstraZeneca Pharmaceutical Co. Ltd. (China; Approval No. HJ20160467).

S‐180 tumor cells (CL‐0201) were kindly provided by Procell Life Science and Technology Co. Ltd.

### 2.2 Preparation Process of TOPs

The preparation process of TOPs is as follows: after humanely euthanizing the tortoises, they are cleaned using a washing machine. The cleaned tortoise meat meets the national safety standards for fresh and frozen animal products in the People's Republic of China (GB 2733) (The food safety standards of the People's Republic of China mentioned in this manuscript can download the specific documents from the following website: http://down.foodmate.net/standard/sort/3/48414.html). The meats from *Cuora trifasciata* , *Mauremys mutica* , and *Chinemys reevesiis* were mixed in a 1:1:1 ratio and then ground using a meat grinder. The ground meat was then transferred to a cooking pot for extraction.

The prepared broth underwent enzymatic hydrolysis. The papain hydrolysate was prepared with the enzymatic hydrolysis conditions set as a material‐to‐water ratio of 1:3, pH 6.5, temperature of 55°C, and hydrolysis time of 4 h, with an enzyme addition of 1.5%. The activity of the papain was determined using the Folin‐phenol method, and the enzymatic activity was 5.3 × 10^4^ U/g. After enzymatic hydrolysis, the clarified solution was stored in a storage tank. The solution was then transferred to an oil–water separation tank for phase separation. The separated liquid was filtered through a ceramic filter. The filtered liquid was concentrated using a single‐effect concentrator. The concentrated liquid was spray‐dried in a 50‐type spray dryer. The solid product obtained from spray drying was vacuum freeze‐dried in a vacuum freeze dryer. After freeze‐drying, the solid was quick‐frozen and then crushed. The resulting powder was portioned into packages. Finally, the packaged powder was sealed for final product packaging (Food Production License No. SC12246010601479).

### 2.3 Nutritional Analysis of TOPs

The nutritional composition of the TOPs sample was analyzed by Guoding Testing Technology Co. Ltd. (Fujian, China). Energy content was assessed in accordance with the National Food Safety Standard for Nutrition Labeling of Prepackaged Foods (GB 28050‐2011). Protein content was determined using Method 1 specified in the National Food Safety Standard for Determination of Protein in Foods (GB 5009.5‐2016), while fat content was measured according to Method 2 of the National Food Safety Standard for Determination of Fat in Foods (GB 5009.6‐2016). Carbohydrate content was analyzed per GB 28050‐2011, and sodium content was quantified using the National Food Safety Standard for Determination of Potassium and Sodium in Foods (GB 5009.91‐2017).

### 2.4 Amino Acid Composition Testing of TOPs

The samples were sent to Beijing Nutrition Source Research Institute Co. Ltd. (Beijing, China) for analysis. The amino acid composition of the TOPs was tested according to the national food safety standard of the People's Republic of China for the determination of amino acids in food (GB 5009.124‐2016).

### 2.5 De Novo Peptide Sequencing by Liquid Chromatography–Tandem Mass Spectrometry (LC–MS/MS)

Peptide samples (< 10 kDa) were subjected to de novo sequencing via LC–MS/MS at Bio‐Tech Pack Technology Company Ltd. (Beijing, China). Briefly, lyophilized peptides were dissolved in Milli‐Q water, reduced with 10 mM DL‐dithiothreitol (DTT) at 56°C for 1 h, and subsequently alkylated in the dark using 50 mM iodoacetamide (IAA) at room temperature for 40 min. After desalting and lyophilization, the samples were reconstituted in 2–20 μL of 0.1% formic acid for LC–MS/MS analysis.

The nanoLC system (Sciex, Framingham, MA, USA) employed an in‐house packed column (150 μm × 17 cm) containing reversed‐phase ReproSil‐Pur C18‐AQ resin (1.9 μm, 100 Å; Dr. Maisch GmbH, Ammerbuch, Germany), coupled to an Ultimate 3000 system (ThermoFisher Scientific, Waltham, MA, USA) and a Q Exactive Hybrid Quadrupole‐Orbitrap Mass Spectrometer (ThermoFisher Scientific). MS/MS data were analyzed using Xcalibur software (Thermo Scientific) with a minimum local confidence threshold of 90% to ensure data reliability.

### 2.6 Bioinformatic Prediction of Anticancer Activity

The prediction of anticancer potential for the peptide molecules was conducted with technical support from Bio‐Tech Pack Technology Company Ltd. (Beijing, China). Briefly, peptide sequences were evaluated for anticancer activity using TriNet (Han et al. 2023), a tri‐fusion neural network‐based tool. Initial bioactivity probability was assessed via the PeptideRanker server (http://distilldeep.ucd.ie/PeptideRanker/), with a threshold score of ≥ 0.8. Sequences exceeding this value were classified as bioactive antitumor peptides.

Molecular docking simulations were performed against key oncogenic targets—BCL‐2 (Protein Data Bank, PDB ID: 2O2F) (Vogler et al. 2025), MDM2 (PDB ID: 1RV1) (Wade et al. 2013), and EGFR (PDB ID: 1M17) (Nyati et al. 2006)—using the CABS‐dock server (https://biocomp.chem.uw.edu.pl/CABSdock/) to evaluate binding affinity and interaction potential. The most stable and predominant binding mode (cluster_1) from each simulation was selected for further analysis. Peptides with a minimum interaction energy < −50 kcal/mol were considered to exhibit strong binding affinity (Halim et al. 2023). For more stringent assessment, peptides with interaction energy < −150 kcal/mol were classified as possessing a super strong binding conformation, while those ranging from −150 to −100 kcal/mol were deemed strong. The stability of peptide–target binding was further evaluated based on the proportion of simulation frames in which the system maintained a high‐affinity binding state.

### 2.7 Concentration Gradient Effect of TOPs

The experiments were approved by the Animal Care and Use Committee of Guangzhou University of Traditional Chinese Medicine (Approval No. 20230618002), and all procedures were conducted in accordance with the ARRIVE guidelines (McGrath and Lilley 2015) and associated guidelines.

The male Kunming mice (KM mice) were housed under a 12‐h light/dark cycle at 26°C. The experiments were conducted in a specific pathogen‐free (SPF) environment.

KM mice, aged 6 weeks, were purchased from Guangzhou University of Traditional Chinese Medicine and housed in an SPF environment. Six mice were kept in each cage. A total of 30 mice were randomly divided into five groups: six mice in the normal control group (NC), six in the model control group (MC), six in the low‐dose group (LD), six in the medium‐dose group (MD), and six in the high‐dose group (HD).

**FIGURE 1:**
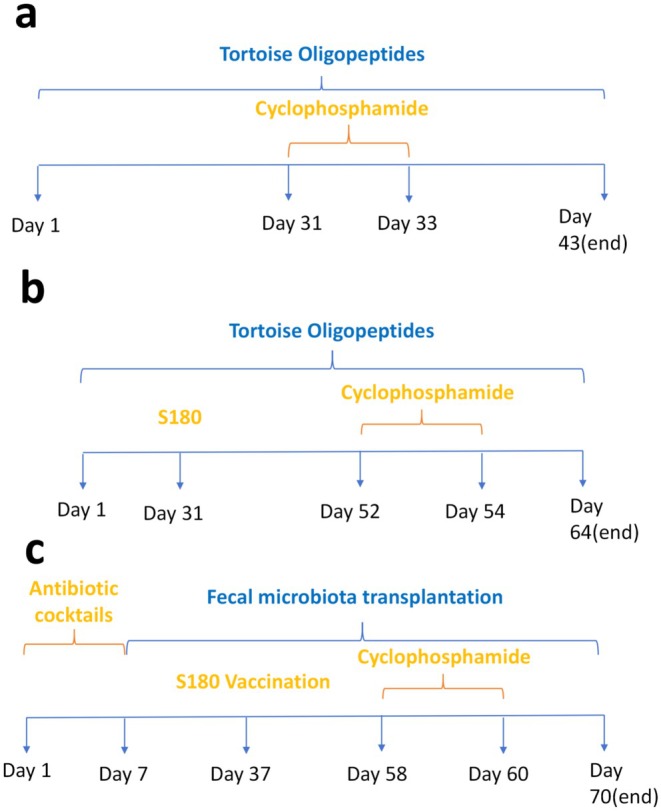
Schematic diagram of experimental procedures. (a) Dose–response assessment of TOPs on CTX‐induced leukopenia. KM mice (*n* = 6 per group) were orally administered either saline (NC and MC groups) or varying doses of TOPs (LD, MD, HD groups: 0.12, 0.24, 0.48 g/mL, respectively) for 30 days. Subsequently, except for the NC group, all mice received intraperitoneal injections of CTX (80 mg/kg) for three consecutive days. Samples were collected 10 days after the first CTX injection. (b) Evaluation of TOPs efficacy in a tumor‐bearing mouse model. Mice were gavaged with saline (TMC and TCTX groups) or high‐dose TOPs (THD group: 0.48 g/mL) for 30 days. On day 31, S180 tumor cells (2 × 10^6^) were inoculated into the right axillary fossa. After confirming tumor establishment, mice received CTX injections (80 mg/kg, i.p. for 3 days) starting on day 54. The experiment concluded 10 days post‐initial CTX injection. (c) Validation of gut microbiota's causal role via FMT. Recipient mice were pre‐treated with an antibiotic cocktail for 1 week to deplete gut microbiota. Following this, they received oral gavage of fecal microbiota suspensions prepared from donor mice (either TCTX or THD groups) for 30 days. The subsequent tumor inoculation, CTX treatment, and sample collection schedule were identical to that in panel (b). CTX, cyclophosphamide; FMT, fecal microbiota transplantation; HD, high‐dose tops; NC, normal control; MC, model control; MD, medium‐dose tops; LD, low‐dose tops; S180, S180 tumor cells; TCTX, tumor‐bearing CTX‐treated; THD, tumor‐bearing high‐dose tops‐treated; TMC, Tumor‐bearing model control; TOPs, tortoise oligopeptides.

Since the maximum solubility of the TOPs is 0.48 g/mL (any higher concentration would clog the gavage needle), this concentration was used for the high‐dose group. The medium‐dose concentration was set at 0.24 g/mL, and the low‐dose concentration at 0.12 g/mL. Each group received a daily gavage of 1 mL/100 g of body weight. The remaining two groups were administered normal saline by gavage at the same dosage (1 mL/100 g) daily. After 30 days of gavage, except NC group, the mice were intraperitoneally injected with CTX at a dose of 80 mg/kg for three consecutive days. Tissues were collected 10 days after the first CTX injection (Figure 1a).

### 2.8 Tumor‐Bearing Mice Experiment

Six‐week‐old male KM mice from Guangzhou University of Chinese Medicine and housed in an SPF environment, with 1 week of adaptive feeding. Three mice were housed per cage. In Animal Experiment 1, we observed a dose–response relationship for TOPs in improving CTX‐induced leukopenia, with high doses of TOPs better increasing White Blood Cell (WBC) counts in mice. Therefore, only the high‐dose TOPs group was set as the intervention group in this experiment.

Forty mice were randomly divided into three groups: 16 mice in the model control group, 12 in the CTX group, and 12 in the high‐dose TOPs group. Tumor‐bearing mice were prepared by injecting 2 × 10^6^ cells (in 200 μL saline) into the axillary fossa of the right foreleg. Ten days later, successful modeling was achieved in 13 mice in the tumor‐bearing model control group (TMC), 7 in the tumor‐bearing CTX group (TCTX), and 7 in the tumor‐ bearing high‐dose TOPs group (THD). The high‐dose group received a daily gavage of 1 mL/100 g of 0.48 g/mL TOPs, while the other two groups received saline at the same dosage (1 mL/100 g) once daily.

After 30 days of gavage, S180 cells were inoculated. Nine days post‐inoculation, tumor size was measured, and 22 days post‐inoculation, CTX was intraperitoneally injected at a dose of 80 mg/kg for three consecutive days. Tissue samples were collected 10 days after the start of CTX injection (Figure 1b).

### 2.9 Fecal Microbiota Transplant (FMT)

Six‐week‐old male KM mice, sourced from Guangzhou University of Chinese Medicine, were acclimated for 1 week under SPF conditions, with three mice per cage. The animals were classified as either donors or recipients. Based on previous results indicating that high‐dose TOPs (THD) improved efficacy and reduced toxicity in CTX‐treated tumor‐bearing mice, donor mice were selected from the TCTX (control group for TOPs) and THD groups. Corresponding recipient mice were assigned to TCTX‐R and THD‐R groups, following the same treatment protocol as described earlier.

FMT was performed on recipient mice (*n* = 8 per group) using a method adapted from a previously published study (Wen et al. 2022). In brief, donor feces were homogenized in 1 mL of sterile PBS in centrifuge tubes, vigorously vortexed, and centrifuged at 800 rpm for 3 minutes. The supernatant, containing the microbiota suspension, was collected, and 200 μL was administered orally to each recipient mouse. Before FMT, all recipients underwent a one‐week pretreatment with 200 μL of an antibiotic cocktail consisting of ampicillin (1 g/L; Beijing Dongge Biotechnology Co. Ltd.), metronidazole (1 g/L; Sichuan Kelun Pharmaceutical Co. Ltd.), neomycin (1 g/L; Hefei Dubang Biopharmaceutical Co. Ltd.), and vancomycin (0.5 g/L; Hainan Poly Pharm. Co. Ltd.).

After 30 days of FMT administration, S180 cells were inoculated into the mice. Tumor size was measured 9 days post‐inoculation. Starting from day 22 after inoculation, mice received intraperitoneal injections of CTX (80 mg/kg) for three consecutive days (as outlined in Figure 1c).

### 2.10 Tumor Size Measurement and Inhibition Rate Calculation

Tumor size was measured using calipers, and the tumor volume was calculated using the formula: length × width^2^/2 (Naito et al. 1986).

The tumor inhibition rate was calculated using the following formula (Ren et al. 2014):

$$\text{Inhibition}\;\left(\%\right)=\left(M-T\right)/M\times 100\%,$$

where M and T represent the mean tumor volume (in cm^3^) of the model control group and treated group, respectively.

### 2.11 Spleen and Thymus Weighing

Based on the recognized correlation between immune activity and the mass of immune organs, the spleen and thymus were weighed, and their relative weights (in mg per 10 g of mouse body weight) were used to calculate the Spleen Index (SI) and Thymus Index (TI), as previously described (Fang et al. 2014; Wiens et al. 2013).

### 2.12 Spleen and Femur Hematoxylin and Eosin (H&E) Staining

After collection, tissue samples were fixed in 10% neutral buffered formaldehyde and promptly delivered to Yuebin Pathology Laboratory for standard H&E staining and section preparation. All slides were randomized and evaluated blindly by Professor Liu from the Department of Pathology, Guangzhou University of Traditional Chinese Medicine.

### 2.13 Blood Collection

Blood samples were collected from mice via retro‐orbital bleeding. A total of 10 μL of whole blood was analyzed immediately using a veterinary blood cell analyzer (Mindray BC‐2800ve). The remaining blood was centrifuged at 3000× *g* for 15 min to isolate serum.

### 2.14 Serum Detection

Serum levels of Interleukin‐4 (IL‐4), Interleukin‐1 beta (IL‐1β), Tumor Necrosis Factor‐alpha (TNF‐α), and Interferon‐gamma (IFN‐γ) were measured using commercial ELISA kits (Nanjing Jiancheng Bioengineering Institute, China) according to the Enzyme‐Linked Immunosorbent Assay (ELISA) manufacturer's instructions. Absorbance was read with a Multiskan Mk3 microplate reader (Thermo Fisher Scientific, Shanghai).

### 2.15 Gut Microbiota Analysis

Fecal samples were sent to OE Biotech Co. Ltd. (Shanghai, China) for sequencing on the Illumina NovaSeq platform. Subsequent bioinformatic analyses—including taxonomic relative abundance, α‐diversity, β‐diversity, and Kyoto Encyclopedia of Genes and Genomes (KEGG) pathway prediction—were performed automatically using the OE Cloud Platform (https://cloud.oebiotech.com/).

### 2.16 Statistical Analysis

The results were analyzed using SPSS 23 software, and data were expressed as mean ± standard error of the mean (s.e.m.). For counting data, use the chi‐square test. For metered data, when data sets were normally distributed with a satisfactory homogeneity of variance, the *p* values were determined using one‐way analysis of variance (ANOVA) with Fisher's least significant difference analysis. Otherwise, the *p* values were determined using the Kruskal–Wallis test. Differences were considered statistically significant at *p* < 0.05.

## 3 Results

### 3.1 Characterization and In Silico Prediction of Antitumor Potential of TOPs

The detected nutrients mainly include protein, as shown in Table 1. Glycine is the amino acid with the highest content in TOPs (16.2 g/100 g), and the other amino acid components are shown in the Data S1.

**TABLE 1:** Nutritional information.

Item	Results	Unit of measurement	Nutrient reference value (%)
Energy	865	kJ/100 g	10%
Protein	28.9	g/100 g	48%
Fat	10.1	g/100 g	17%
carbohydrate	0	g/100 g	0%
sodium	484	mg/100 g	24%

*Note:* The measured water content was 4.1 g/100 g; the measured ash content was 56.5 g/100 g.

**FIGURE 2:**
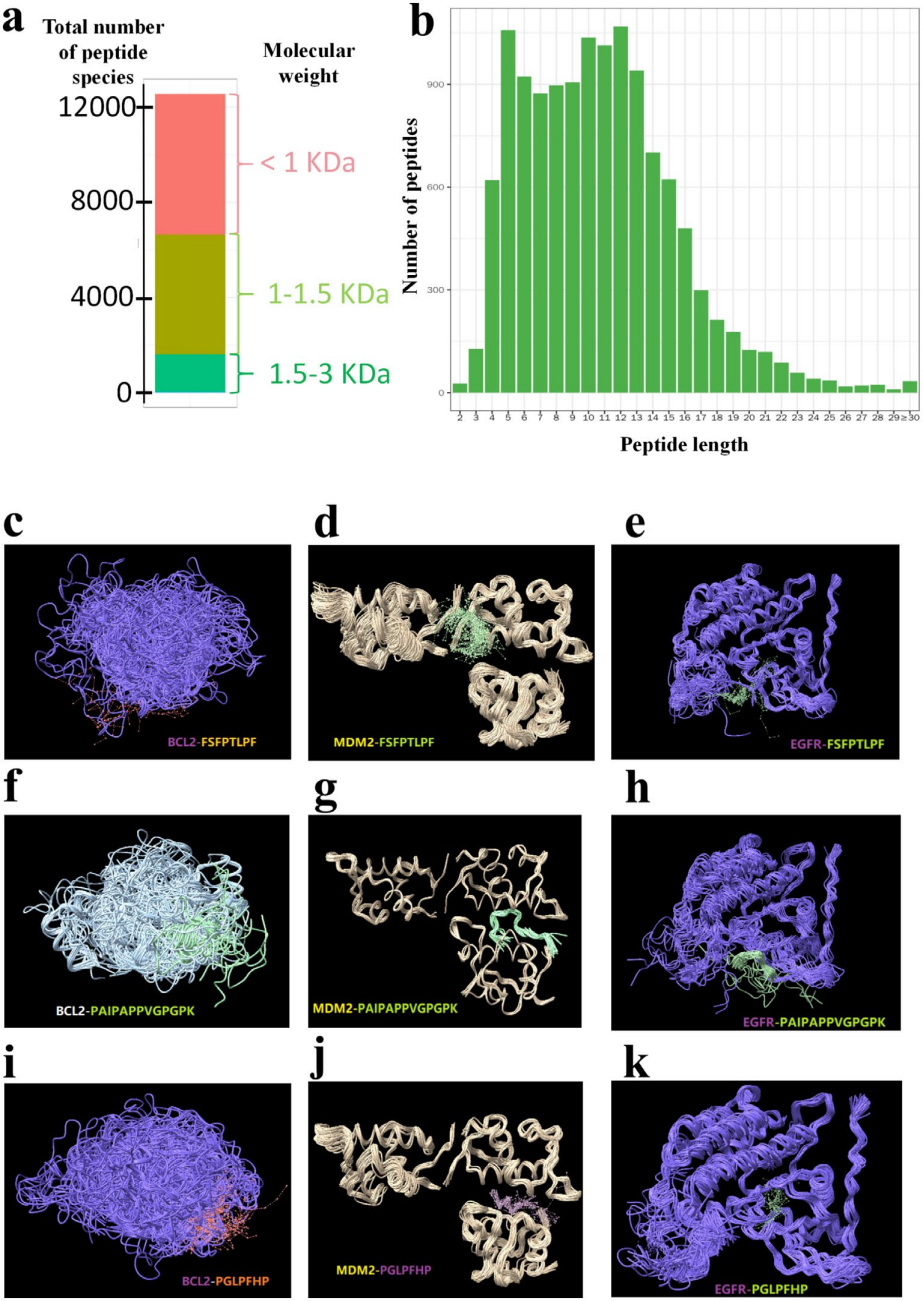
Characterization and in silico analysis of TOPs. Distribution of (a) Relative molecular weight and (b) Peptide length of TOPs. Molecular docking simulations between: (c) BCL‐2 and FSFPTLPF; (d) MDM2 and FSFPTLPF; (e) EGFR and FSFPTLPF; (f) BCL‐2 and PAIPAPPVGPGPK; (g) MDM2 and PAIPAPPVGPGPK; (h) EGFR and PAIPAPPVGPGPK; (i) BCL‐2 and PGLPFHP; (j) MDM2 and PGLPFHP; (k) EGFR and PGLPFHP. TOPs: Tortoise oligopeptides.

A total of 12,561 small molecular peptides were identified in the TOPs sample. The peptide mainly had molecular weights of no more than 5 kDa (Figure 2a). The peptide lengths were predominantly between 4 and 15 amino acids (Figure 2b). The types of identified small molecular peptides and their relative abundance in the sample are summarized in the Data S2.

We screened 4840 small molecule peptides with anti‐tumor characteristics in the 12,561 contained in TOPs. From the 4840 small molecule peptides, 678 small molecule peptides with anti‐tumor biological activity were screened with a threshold of 0.8. These 678 small molecule peptides with anti‐tumor biological activity accounted for about 6.56% of the total amount of TOPs (Data S3). We selected the 10 small molecule peptides with the highest content (accounting for about 3.10% of the total TOPs) and some of the peptides closely related to BCL‐2, MDM2, and EGFR as docking targets for molecular docking simulation. The results show that although these 10 small molecule peptides have good affinity with anti‐tumor targets (minimum interaction energy < −50 kcal/mol) in the process of simulated molecular docking, according to our stricter screening criteria, small molecule peptides such as IPGPF or LPGPF cannot form a stable strong binding conformation with BCL‐2, MDM2, or EGFR. PAIPAPPVGPGPK, FSFPTLPF, and PGLPFHP can form a stable and strong binding conformation with BCL‐2, MDM2, and EGFR at the same time (Figure 2c–k). It suggests that these three small molecule peptides can produce antitumor effects by inhibiting multiple tumor targets. At the same time, other small molecule peptides, such as GPYGL can only form stable strong binding structures with one or two of the three anti‐tumor targets detected. The specific situation is shown in Table 2.

**TABLE 2:** Molecular docking of the 10 potential anti‐tumor small molecule peptides with the highest content.

Peptide	Percentage of content (%)	Minimum interaction energy (kcal/mol) & proportion of strong bonding conformation (%)					
		Bcl‐2		MDM2		EGFR	
LPGPF	0.8028214	−73.6	0	−10.54	0	−67	0
IPGPF	0.8028214	−57	0	−71.4	0	−73.35	0
PAIPAPPVGPGPK	0.450021	−138.6	17	−221	87.8	−166.1	44.8
FSFPTLPF	0.2635981	−112.1	0.3	−115	2.4	−124.4	1.1
PGPMGPRGPA	0.2225048	−84.7	0	−113.8	2.6	−136.15	12.2
GPVGPSGPPGIP	0.1262865	−142.4	34.6	−189.8	72.7	−98.2	0
GLPFHP	0.1112524	−94.6	0	−99.6	0	−71.6	0
GPYGL	0.1092479	−99.2	0	−101	0.6	−51.8	0
SGPPGLPGPIGLPGDPG	0.1042365	−68.3	0	−188.3	45.3	−153.95	27.7
PGLPFHP	0.1022319	−132.15	30.5	−139	70.2	−115	10

*Note:* Minimum interaction energy: The strongest binding force observed in the entire simulation, which is used to evaluate the binding strength; the lower the value, the higher the binding strength. Proportion of strong bonding conformation: The higher the proportion of frames accounted for by the system maintaining a very strong bonding state (Minimum interaction energy ≤ −100 kcal/mol) during the entire simulation time, the more stable the bonding system.

### 3.2 TOPs Alleviate CTX‐Induced Leukopenia and Immune Dysfunction

**FIGURE 3:**
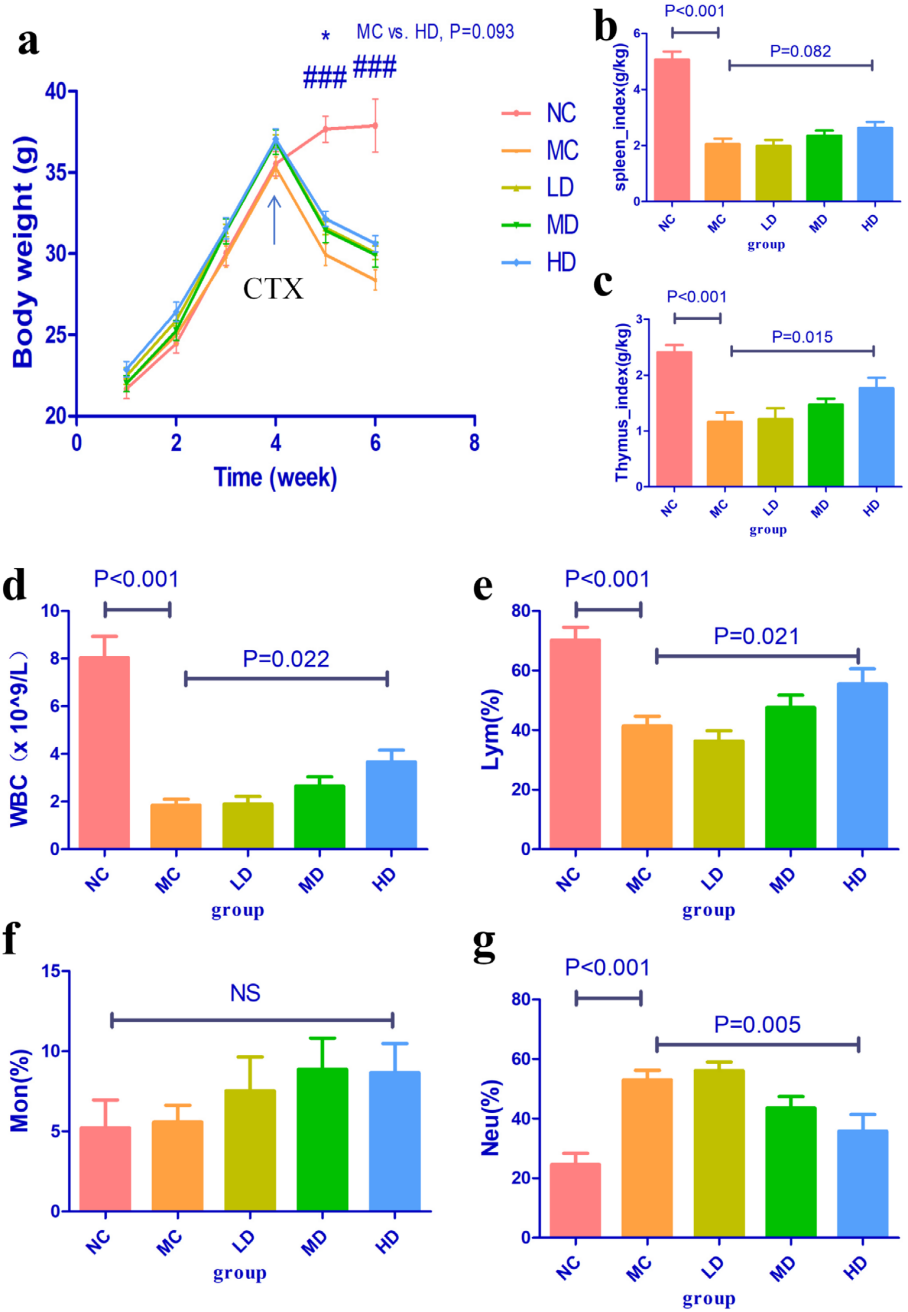
Effects of TOPs on CTX‐induced leukopenia in non‐tumor‐bearing mice. (a) Body weight changes, (b) Spleen index, (c) Thymus index, (d) WBC count, (e) Lymphocyte percentage, (f) Monocyte percentage, (g) Neutrophil percentage. Data are presented as mean ± s.e.m.; *n* = 6 per group. *p < 0.05, MC vs. HD group; ^###^ *p* < 0.001, NC vs. MC group. CTX, cyclophosphamide. TOPs, tortoise oligopeptides.

**FIGURE 4:**
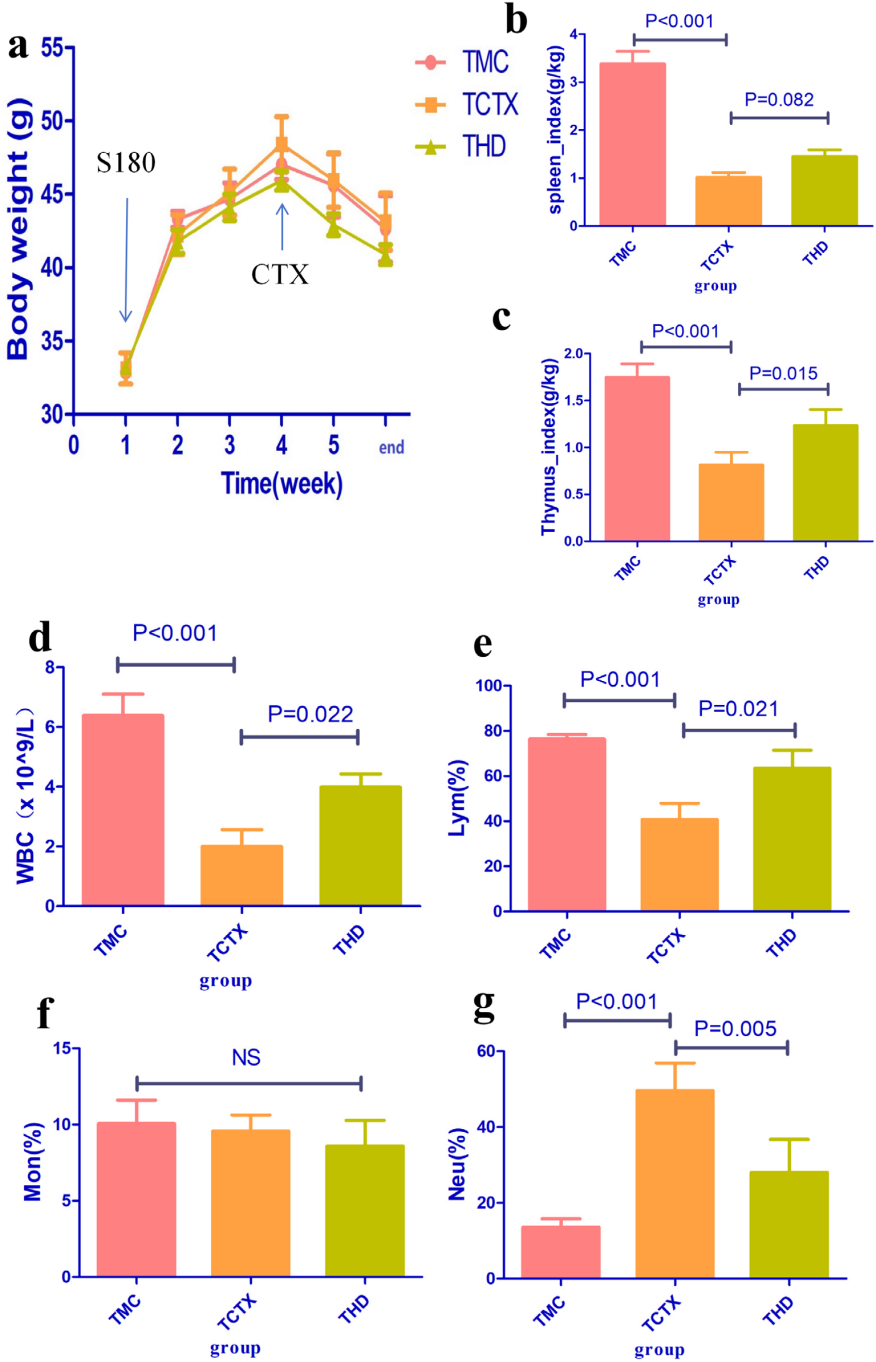
Effects of TOPs on tumor‐bearing mice treated with CTX. (a) Body weight changes, (b) spleen index, (c) thymus index, (d) WBC count, (e) lymphocyte percentage, (f) monocyte percentage, (g) neutrophil percentage. *n* = 6 per group. CTX, cyclophosphamide. TOPs, tortoise oligopeptides. S180, S180 tumor cells.

In the non‐tumor‐bearing mice experiment, compared to the NC group, the body weight of mice in all other groups significantly decreased. After CTX treatment, during the fifth and sixth weeks of the experimental period, the body weight of mice in the HD group was significantly higher than the MC group (HD vs. MC group, *p* < 0.05, Figure 3a). In the tumor‐bearing mice experiment, there was no significant difference in body weight among the groups (*p* > 0.05, Figure 4a).

In experiments with both non‐tumor‐bearing mice and tumor‐bearing mice, CTX significantly reduced the SI and TI in mice. Pre‐intervention with TOPs was able to upregulate both the SI and TI in these mice (Figures 3b,c and 4b,c).

In both non‐tumor‐bearing and tumor‐bearing mouse models, CTX significantly reduced leukocyte counts and lymphocyte percentages. However, pre‐treatment with TOPs effectively increased leukocyte counts and lymphocyte percentages in these mice (Figures 3d,e and 4d,e).

**FIGURE 5:**
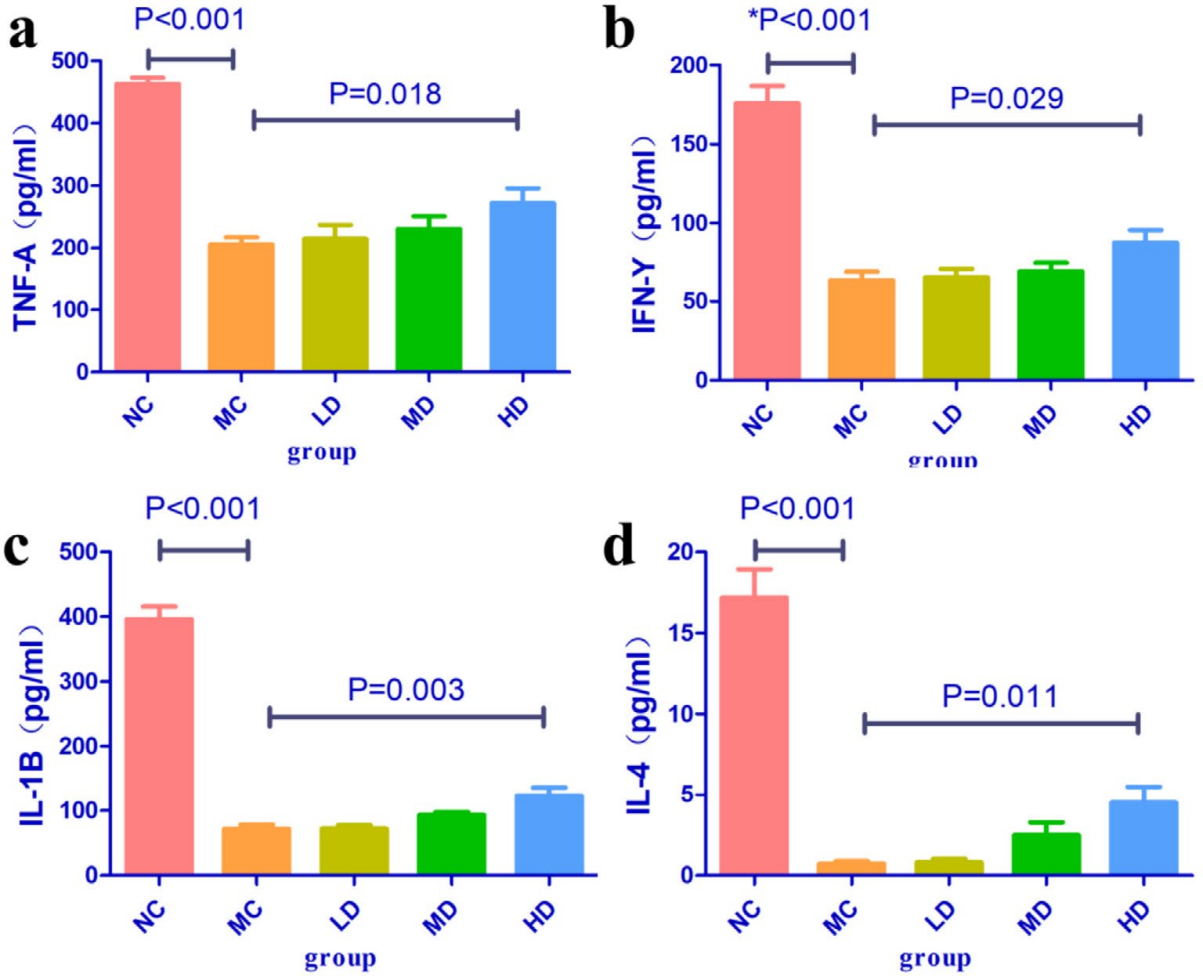
Serum cytokine levels in mice with CTX‐induced leukopenia treated with TOPs. (a) TNF‐α, (b) IFN‐γ, (c) IL‐1β, (d) IL‐4. *n* = 6 per group. CTX, cyclophosphamide. TOPs, tortoise oligopeptides.

In experiments with non‐tumor‐bearing mice, CTX significantly reduced the serum levels of IL‐4, IL‐1β, TNF‐α, and IFN‐γ in mice, while TOPs could upregulate the serum levels of IL‐4, IL‐1β, TNF‐α, and IFN‐γ (Figure 5).

### 3.3 TOPs Mitigate CTX‐Induced Pathological Damage in Spleen and Bone Marrow

**FIGURE 6:**
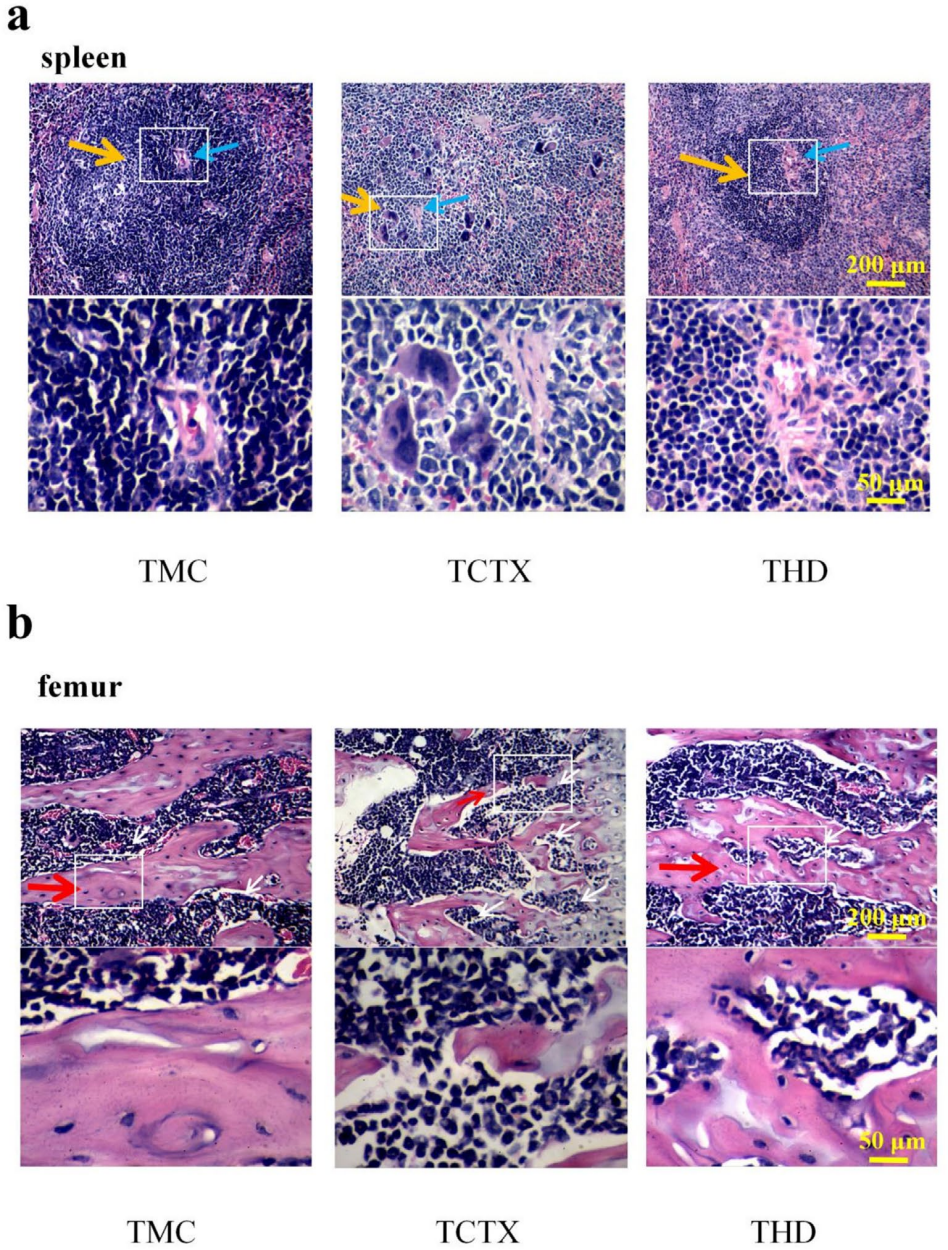
Histopathological analysis of spleen and bone marrow in tumor‐bearing mice treated with CTX and TOPs. (a) Spleen sections: orange arrows indicate splenic corpuscles; blue arrows indicate periarterial lymphatic sheaths. Total magnification: 100×; scale bar = 200 μm. Inset: 4× magnification of boxed area; scale bar = 50 μm. (b) Bone marrow sections: red arrows indicate bone trabeculae; white arrows indicate resorption pits. Total magnification: 100×; scale bar = 200 μm. Inset: 4× magnification of boxed area; scale bar = 50 μm. CTX, cyclophosphamide. TOPs, tortoise oligopeptides.

As shown in Figure 6a: Tumor‐bearing Model Control Group: Splenic white pulp exhibited well‐defined splenic corpuscles without atrophy of the periarterial lymphatic sheaths. Germinal centers within lymphatic follicles were clearly visible. Red pulp showed no lymphocyte proliferation or increases in megakaryocytes or macrophages.

Tumor‐bearing CTX Group: White pulp displayed significant atrophy and reduced numbers of splenic corpuscles. Periarterial lymphatic sheaths and surrounding lymphatic nodules were markedly shrunken, with substantial size reduction. Some splenic corpuscles exhibited indistinct structural organization. Red pulp revealed mild lymphocyte proliferation alongside increased multinucleated giant cells and macrophages.

Tumor‐bearing High‐dose Group: White pulp contained clearly visible splenic corpuscles with prominent periarterial lymphatic sheaths. Lymphatic follicles maintained well‐organized architecture without proliferation of multinucleated giant cells or macrophages. Red pulp demonstrated no significant lymphocyte proliferation.

As shown in Figure 6b: Tumor‐bearing Model Control Group: Bone trabeculae appeared thick and continuous, forming a well‐organized reticular framework. Trabecular surfaces exhibited no increase in osteoclasts or resorption pits. The bone marrow cavity showed no evidence of elevated osteoclast activity.

Tumor‐bearing CTX Group: Bone trabeculae displayed significant reductions in both thickness and number, accompanied by poor continuity and frequent disruptions. Osteoclast numbers increased around trabeculae, with elevated resorption pit formation on their surfaces. The bone marrow cavity also exhibited increased osteoclast presence.

Tumor‐bearing High‐dose Group: Bone trabeculae maintained normal thickness and continuity without significant interruptions. Trabecular surfaces showed no significant increase in osteoclasts or resorption pits. The bone marrow cavity contained only minimal osteoclast presence.

### 3.4 TOPs Enhance the Antitumor Efficacy of CTX in Tumor‐Bearing Mice

During the intervention, in accordance with animal ethics requirements, 7 mice in the model control group, 1 in the CTX group, and 1 in the high‐dose tortoise peptide group were euthanized due to tumor diameters exceeding 20 mm. A chi‐square test was used to compare different interventions affected the mortality rate of mice euthanized due to excessive tumor size. As shown in Table 3, there was a trend of difference between the intervention groups, with *p* = 0.093.

**TABLE 3:** Tumor‐bearing mice were euthanized due to excessive tumor size.

	TMC	TCTX	THD	Total
survival	6	6	6	18
euthanasia	7	1	1	9
Total	13	7	7	27

*Note: X* ^2^ = 4.747; *p* = 0.093.

**FIGURE 7:**
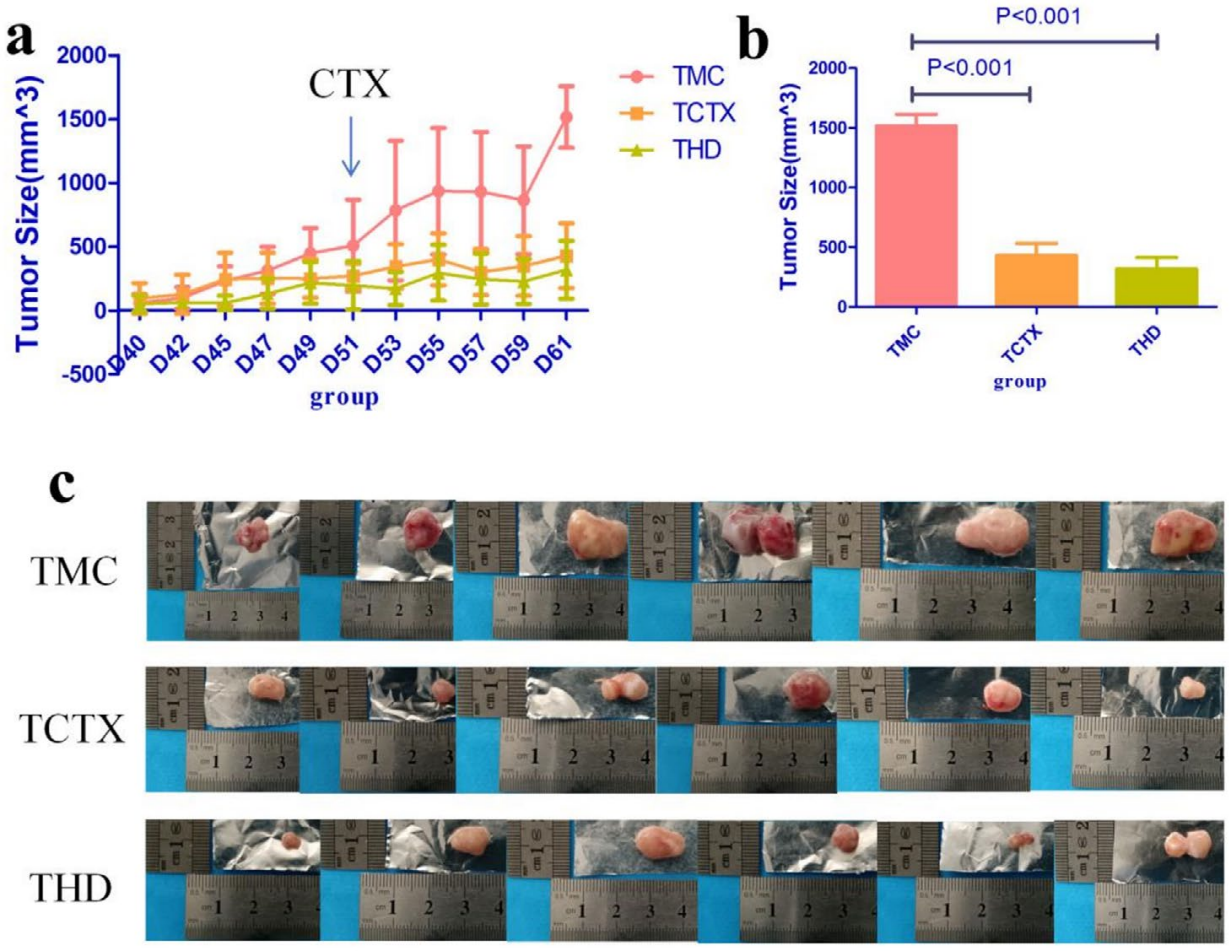
Antitumor effects of TOPs in CTX‐treated tumor‐bearing mice: (a) Tumor volume changes over time, (b) final tumor volume comparison, (c) representative images of excised tumors from each group. CTX, cyclophosphamide. TOPs, tortoise oligopeptides.

As shown in Figure 7a, the tumors of the mice in each group gradually grew after being inoculated with S180 cells in the armpit. Compared with the TMC group, the tumor volume of the THD and TCTX groups was significantly reduced after receiving CTX intervention (*p* < 0.05).

In tumor‐bearing mouse experiments, the tumor inhibition rate of CTX alone was 71.7%, while the combination of TOPs and CTX achieved a tumor inhibition rate of 78.94%. The tumor volume growth in each group of mice is presented in Figure 7c. Although the mean tumor volume in the THD group (319.17 mm^3^) was smaller than that of the TCTX group (428.83 mm^3^) in the final measurement, there was no statistically significant difference between the two groups (*p* = 0.442) (Figure 7b).

### 3.5 TOPs Modulate Gut Microbiota Composition and Function

**FIGURE 8:**
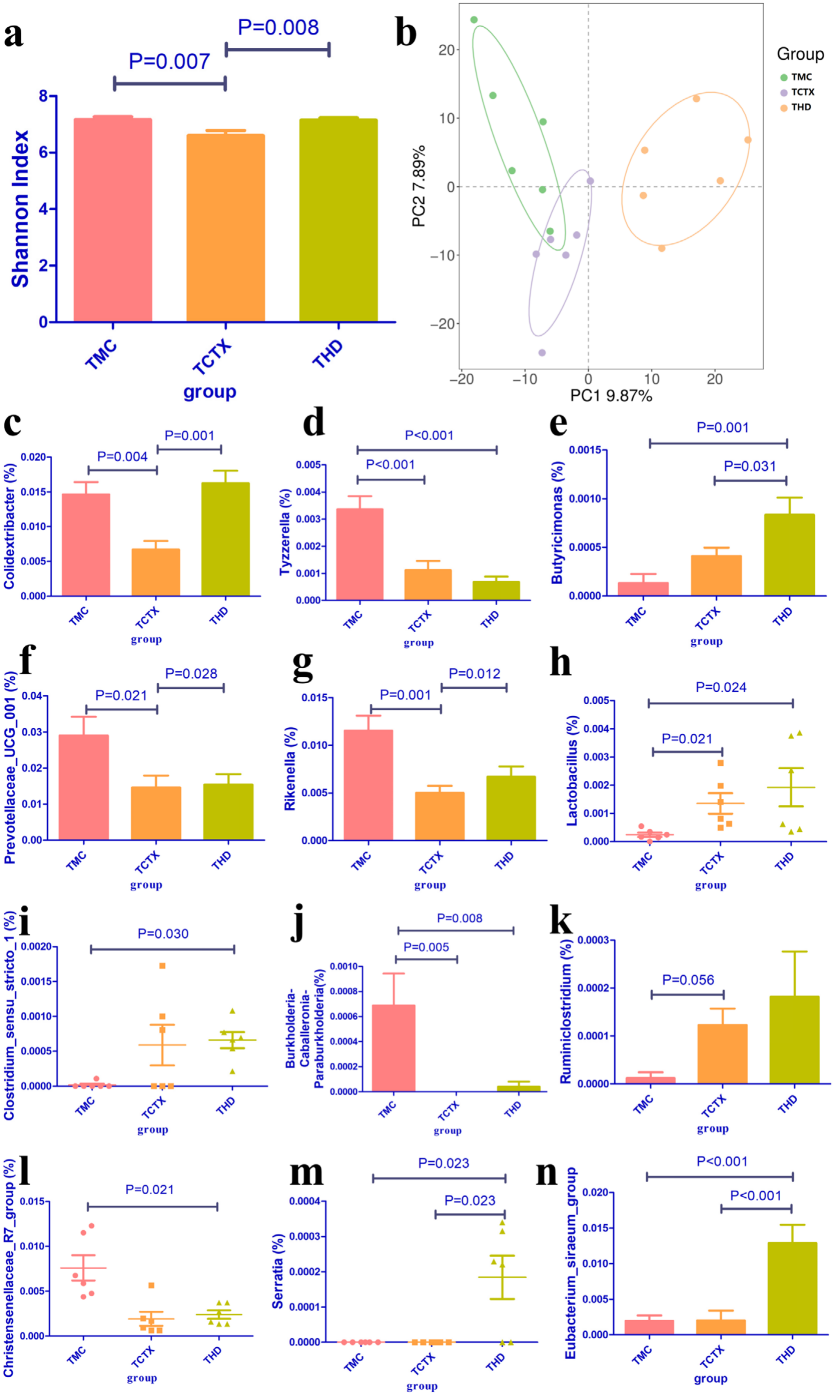
Gut microbiota analysis in tumor‐bearing mice treated with CTX and TOPs. (a) Shannon diversity index, (b) β‐diversity PCA plot, (c–n) Relative abundance of indicated bacterial genera. *n* = 6 per group. CTX, cyclophosphamide. TOPs, tortoise oligopeptides.

**FIGURE 9:**
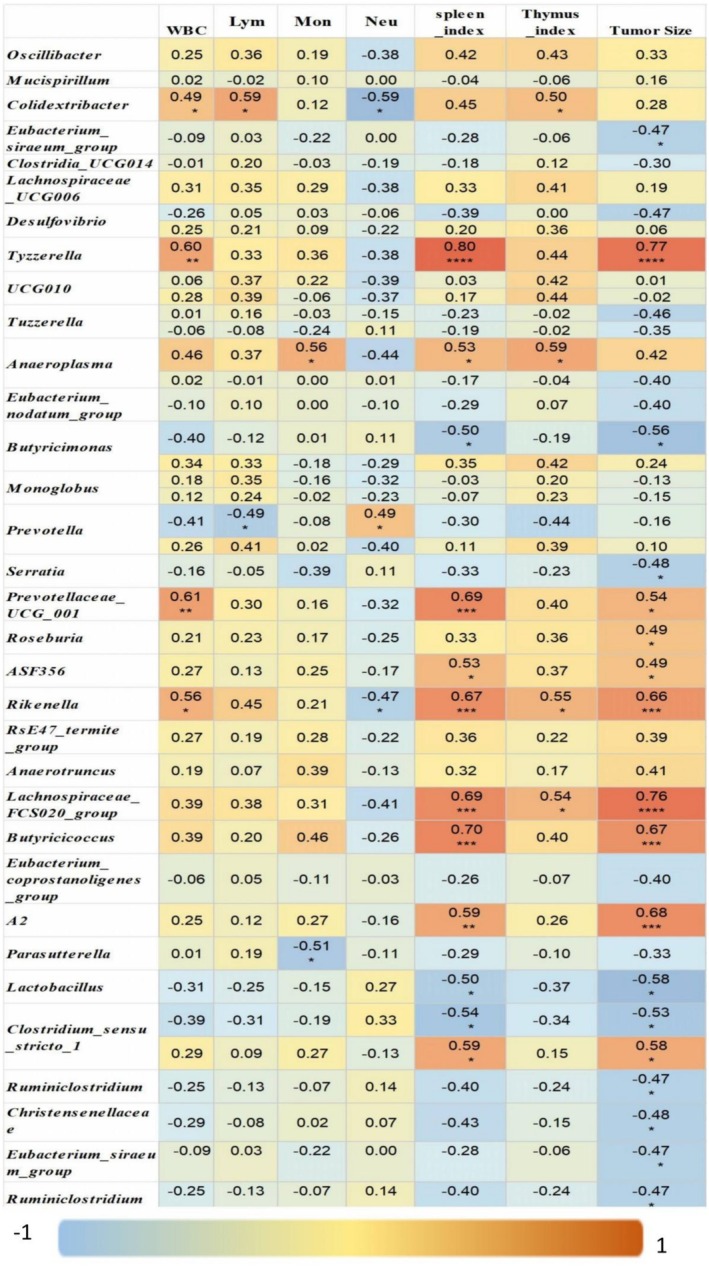
Correlation heatmap between gut microbiota genera and immunological parameters in tumor‐bearing mice. *p < 0.05, **p < 0.01, ***p < 0.001.

CTX significantly reduced the Shannon index, a measure of α‐diversity, in tumor‐bearing mice, whereas treatment with TOPs restored it (THD vs. TCTX, *p* < 0.05; Figure 8a). β‐Diversity analysis further revealed distinct structural differences in microbial communities among groups (Figure 8b). Correlation analyses identified several genera significantly associated with immunological and tumor parameters. For instance, *Colidextribacter*, *Tyzzerella*, *Rikenella*, *Prevotellaceae_UCG_001*, *Lachnospiraceae_FCS020_group*, *Butyricicoccus*, *A2*, and *Burkholderia‐Caballeronia‐Paraburkholderia* showed positive correlations with leukocyte counts, SI, TI, and/or tumor volume (Figures 8c,d,f,g,j and 9). Conversely, *Eubacterium_siraeum_group*, *Serratia*, *Ruminiclostridium*, *Christensenellaceae_R7_group*, *Butyricimonas*, *Lactobacillus*, and *Peptococcus* were negatively correlated with tumor size and/or immune organ indices (Figures 8e,h,k–n and 9). *Anaeroplasma* was positively correlated with monocyte percentage and immune organ indices, while *Roseburia* and *ASF356* correlated positively with tumor size (Figure 9).

**FIGURE 10:**
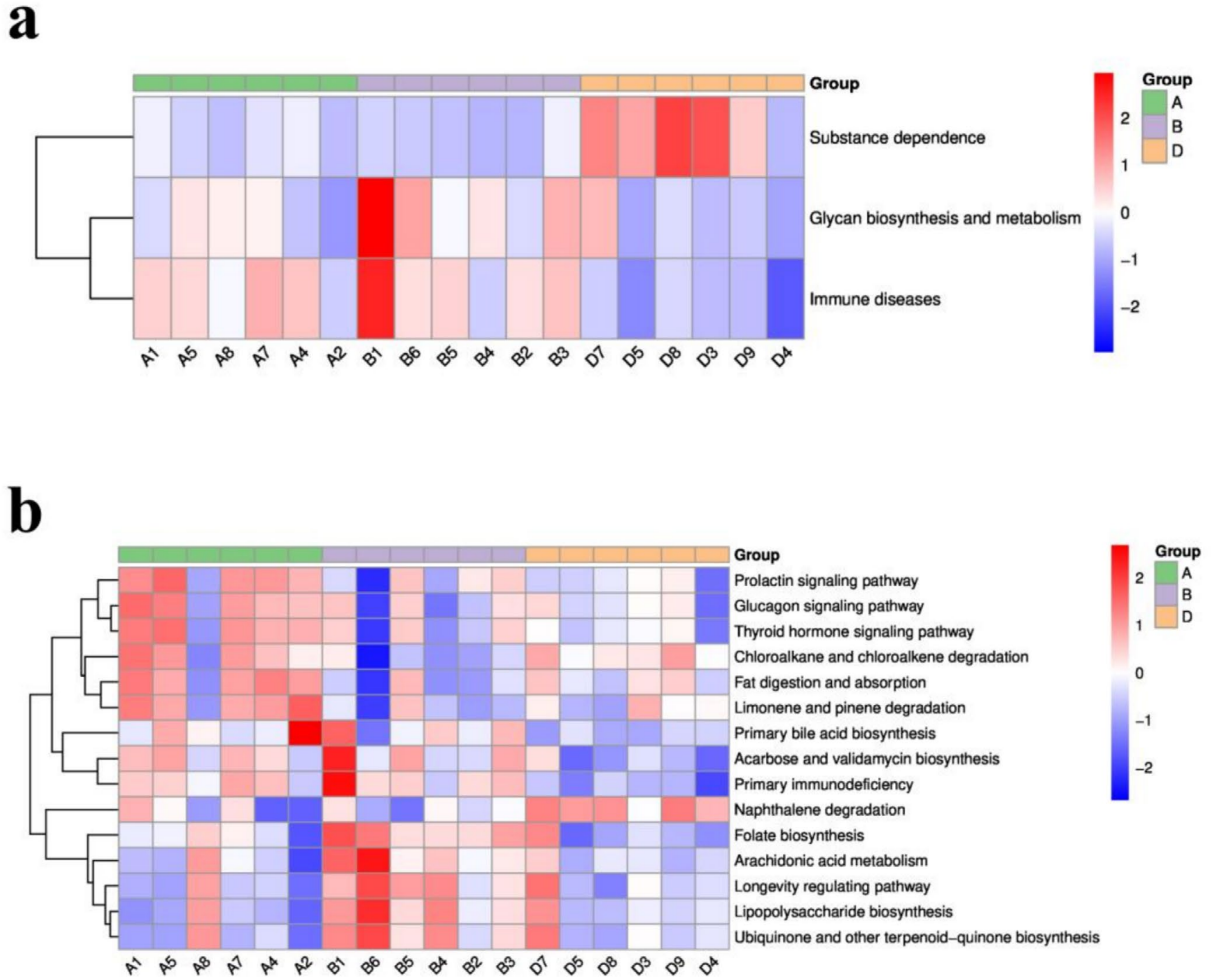
Functional prediction of gut microbiota via KEGG pathway analysis: (a) KEGG level 2 and (b) level 3 pathway differences between groups. KEGG, kyoto encyclopedia of Genes and Genomes.

Functional analysis using KEGG pathway enrichment revealed group differences in Level 2 pathways, including “Substance Dependence,” “Glycan Biosynthesis and Metabolism,” and “Immune Diseases” (Figure 10a). At Level 3, differential abundance was observed in the “Prolactin Signaling Pathway” and “Primary Immunodeficiency” (Figure 10b).

### 3.6 FMT Confirms the Causal Role of Gut Microbiota in Myeloprotection but Not in Antitumor Efficacy

During the intervention, in accordance with animal ethics requirements, 2 mice in the TCTX‐R group and 1 in the THD‐R group were euthanized due to tumor diameters exceeding 20 mm.

**FIGURE 11:**
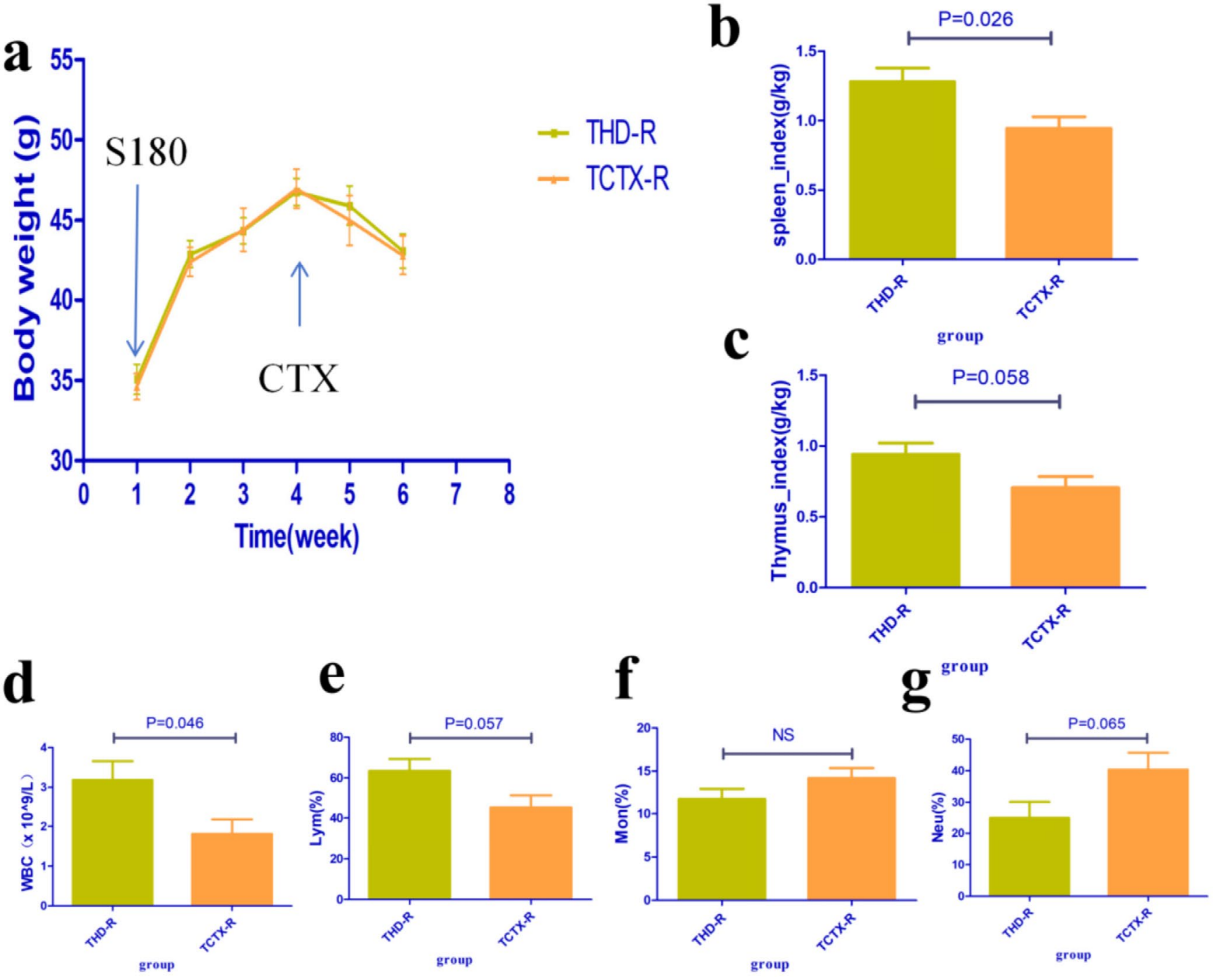
Effects of fecal microbiota transplantation from TOPs‐treated donors on hematological parameters in tumor‐bearing mice. (a) Body weight changes, (b) Spleen index, (c) Thymus index, (d) WBC count, (e) Lymphocyte percentage, (f) Monocyte percentage, (g) Neutrophil percentage. *n* = 6 for FMT‐CTX group, *n* = 7 for FMT‐HD group. CTX, cyclophosphamide; S180, S180 tumor cells. FMT, fecal microbiota transplantation; TOPs, tortoise oligopeptides.

Compared to TCTX‐R, the THD‐R group exhibited improvements in SI, TI, WBC count, and lymphocyte percentage (Figure 11b–e).

**FIGURE 12:**
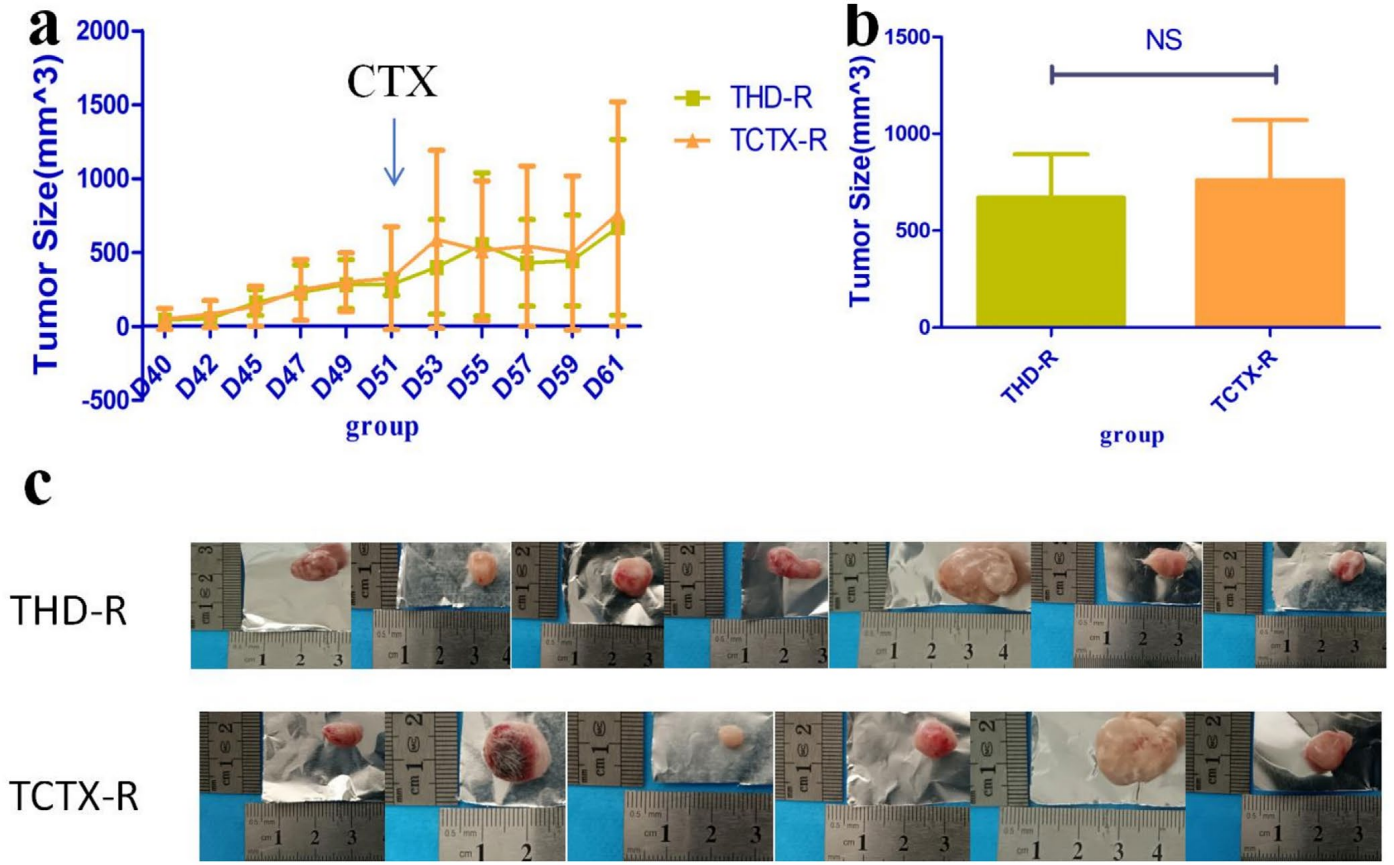
Tumor growth in recipient mice following FMT from TOPs‐treated donors: (a) Tumor volume changes, (b) final tumor volume, (c) representative images of excised tumors. *n* = 6 for FMT‐CTX group, *n* = 7 for FMT‐HD group. CTX, cyclophosphamide; FMT, fecal microbiota transplantation.

However, compared to the TCTX‐R group, mice in the THD‐R group showed no significant differences in body weight change or tumor size (Figure 12a–c).

## 4 Discussion

The primary challenge in CTX chemotherapy lies in managing its hematologic toxicity, particularly leukopenia, without compromising its antitumor efficacy (Zhou et al. 2023). While growth factors like rhG‐CSF address myelosuppression, concerns about their potential to promote tumor growth highlight the need for alternative strategies (Sun et al. 2018; Wang et al. 2022). Based on the traditional use of tortoise meat for enhancing immunity and its nutritional value, we hypothesized that TOPs could serve as a multifaceted adjuvant to achieve this dual goal. Our study provides a mechanistic dissection, demonstrating that TOPs alleviate CTX‐induced leukopenia primarily through gut microbiota‐dependent immunomodulation, while concurrently enhancing CTX's antitumor effect via direct actions of specific bioactive peptides.

### 4.1 TOPs Ameliorate CTX‐Induced Hematologic and Immune Toxicity Through Multilevel Immunonutritional Support

Our initial investigation focused on the potential of TOPs to mitigate the primary adverse effect of CTX. We first established a dose‐dependent rescue of CTX‐induced leukopenia and lymphopenia by TOPs (Figures 3 and 4). This peripheral recovery was not an isolated phenomenon but was underpinned by a systemic restoration of immune homeostasis. The upregulation of spleen and thymus indices (Figures 3b,c and 4h,i) indicated a recovery of central and peripheral immune organs, which was further corroborated by the amelioration of pathological damage in the spleen and bone marrow (Figure 7). The attenuation of splenic corpuscle atrophy and bone trabecular degeneration suggested that TOPs supported the architecture of tissues responsible for hematopoiesis and immune cell maturation. Delving into the molecular mechanism, we found that TOPs significantly restored the serum levels of key cytokines (IL‐4, IL‐1β, TNF‐α, IFN‐γ; Figure 6), which are critical for lymphocyte differentiation, activation, and innate immune responses (Chen et al. 2023). Thus, this first line of evidence paints a coherent picture: TOPs provide nutritional and immunomodulatory support that acts at cellular, organ, and molecular levels to counteract CTX‐induced myelosuppression and immune dysfunction.

### 4.2 The Antitumor Enhancement by TOPs Is Mediated by Direct‐Action Bioactive Peptides, Not Gut Microbiota

Having established the myeloprotective effects of TOPs, we next investigated whether this came at the cost of compromising CTX's antitumor efficacy. Surprisingly, the combination of TOPs and CTX exhibited a higher tumor inhibition rate than CTX alone (78.94% vs. 71.7%), though the difference in final tumor volume was not statistically significant (Figure 7). This intriguing trend suggested a potential synergistic effect rather than interference.

We initially postulated that this enhanced efficacy could be an indirect result of the improved immune system or the modulated gut microbiota. To test this hypothesis, we employed FMT. This experiment provided a pivotal insight:

While FMT from TOPs‐treated donors successfully transferred the protective effect against leukopenia to recipient mice (Figure 11b–e), it did not replicate the antitumor effect (Figure 12a–c). This clear dissociation demonstrated unequivocally that the gut microbiota is necessary and sufficient for the myeloprotective (“toxicity‐reducing”) effect of TOPs, but is not involved in its antitumor (“efficacy‐enhancing”) effect.

This compelled us to search for an alternative, microbiota‐independent mechanism. We turned to the intrinsic properties of TOPs. Bioinformatic analysis and molecular docking simulations identified several peptides within TOPs, most notably PAIPAPPVGPGPK, FSFPTLPF, and PGLPFHP, with high binding affinity and stability against key oncogenic targets (BCL‐2, MDM2, EGFR; Figure 2, Table 2) (Nyati et al. 2006; Vogler et al. 2025; Wade et al. 2013). This suggests that these bioactive peptides could directly inhibit tumor survival pathways, providing a plausible direct‐acting mechanism for the observed trend toward enhanced efficacy alongside CTX. Therefore, this second line of evidence, crucially guided by the FMT experiment, allows us to disentangle the dual effects of TOPs: gut microbiota mediates the mitigation of toxicity, while specific peptides within TOPs are responsible for the enhancement of chemotherapy efficacy.

### 4.3 Gut Microbiota Modulation Is a Key Mechanism for Myeloprotection

The FMT experiment confirmed the causal role of gut microbiota in the myeloprotective effects of TOPs. We further characterized this modulation by showing that TOPs reversed the CTX‐induced decline in gut microbial α‐diversity (Shannon index, Figure 8a) and altered its overall community structure (β‐diversity, Figure 8b). Correlation analysis revealed that genera whose abundance was promoted by TOPs, such as *Colidextribacter* and *Rikenella*, were positively associated with leukocyte counts and immune indices (Figures 8 and 9). Furthermore, functional prediction via KEGG pathway analysis indicated that TOPs influenced microbial functions related to “Immune Diseases” and “Primary Immunodeficiency” (Figure 10). This final line of evidence solidifies the role of gut microbiota: TOPs shape a microbial environment that is conducive to immune recovery, thereby indirectly mediating the protection against CTX‐induced hematologic toxicity.

### 4.4 Limitations and Future Perspectives

Our study has several limitations. The use of a blended oligopeptide preparation from three tortoise species, while justified by its alignment with traditional practices and its advantage in ensuring compositional consistency across batches, prevents attribution of the observed effects to any single species. Future studies should compare the efficacy of oligopeptides derived from each species individually to identify the most potent source and potential synergistic effects. Furthermore, the antitumor potential of the identified peptides, such as PAIPAPPVGPGPK, requires validation in vivo using synthetic peptides to confirm their direct functional role. The precise mechanisms by which the modulated gut microbiota and its metabolites (e.g., Short‐Chain Fatty Acids, SCFAs) influence myelopoiesis also warrant further investigation.

## 5 Conclusion

In conclusion, through a stepwise investigative approach, we have deciphered the dual mechanisms of TOPs in augmenting CTX chemotherapy. We first established their role in alleviating leukopenia via multi‐level immunonutritional support. Subsequently, by employing a critical FMT experiment, we dissociated the dual effects, demonstrating that the enhancement of antitumor efficacy is independent of gut microbiota and is likely attributable to direct‐acting bioactive peptides targeting core oncoproteins. Our findings position TOPs not only as a promising candidate for a specialized medical food to improve chemotherapy tolerance and outcomes but also provide a robust framework for mechanistically understanding the function of complex natural product derivatives.

## Author Contributions

**Zhuotao Fu:** conceptualization (equal), methodology (equal), writing – original draft (equal). **Tian Yu:** formal analysis (equal). **Cong Meng:** funding acquisition (equal). **Guoni Zhang:** formal analysis (equal). **Shaojun Huang:** writing – review and editing (equal). **Yuping Li:** data curation (equal). **Linchun Fu:** visualization (equal). **Zhitong Deng:** funding acquisition (equal), visualization (equal).

## Conflicts of Interest

The authors declare no conflicts of interest.

## Supporting information


**Data S1:** fsn371078‐sup‐0001‐Supplement1.docx.

**Data S2:** fsn371078‐sup‐0002‐Supplement2.docx.

**Data S3:** fsn371078‐sup‐0003‐Supplement3.docx.

## Data Availability

Data for this article, including raw data, are available at Figshare at https://doi.org/10.6084/m9.figshare.30188821.
