# Supplementary material for: Tortoise Oligopeptides Augment Cyclophosphamide's Antitumor Activity Through Dual Modulation of Therapeutic Efficacy and Hematologic Toxicity
Source: Food Sci Nutr. 2025 Oct 16;13(10):e71078. doi: 10.1002/fsn3.71078 (PMC12531418; doi:10.1002/fsn3.71078)
Supplement: Supplementary file 1 — Data S1: fsn371078‐sup‐0001‐Supplement1.docx. [file FSN3-13-e71078-s002.docx]

Supplement 1 Amino Acid Composition

| Amino acid | Content(g/100g) |
| --- | --- |
| Asp | 4.71 |
| Thr | 2.03 |
| Sr | 3.62 |
| Gu | 8.76 |
| Gly | 16.2 |
| Ala | 6.55 |
| Val | 1.81 |
| Met | 0.61 |
| Ile | 1.43 |
| Leu | 2.68 |
| Tyr | 0.61 |
| Phe | 1.84 |
| Lys | 3.05 |
| His | 1.00 |
| Arg | 6.15 |
| Pro | 8.95 |
| Trp | 0.078 |
| Cystine | 0.19 |

Specific amino acids and corresponding weights contained in each 100g of tortoise oligopeptides
