# Supplementary material for: Tortoise Oligopeptides Augment Cyclophosphamide's Antitumor Activity Through Dual Modulation of Therapeutic Efficacy and Hematologic Toxicity
Source: Food Sci Nutr. 2025 Oct 16;13(10):e71078. doi: 10.1002/fsn3.71078 (PMC12531418; doi:10.1002/fsn3.71078)
Supplement: Supplementary file 2 — Data S2: fsn371078‐sup‐0002‐Supplement2.docx. [file FSN3-13-e71078-s001.docx]

| **Peptide** | **-10lgP** | **Mass** | **Length** | **ppm** | **m/z** | **z** | **RT** | **AreaSample1** |
| --- | --- | --- | --- | --- | --- | --- | --- | --- |
| GFDFSFLPQPPQE | 44.78 | 1507.6983 | 13 | 0.7 | 754.85693 | 2 | 53.6034 | 63600000 |
| DFSFLPQPPQE | 44.42 | 1303.6084 | 11 | 1.1 | 652.81219 | 2 | 48.2514 | 333000000 |
| PSGGFDFSFLPQPPQE | 43.94 | 1748.8046 | 16 | -0.6 | 875.409 | 2 | 52.315 | 359000 |
| FSFLPQPPQE | 43.8 | 1188.5815 | 10 | 1.4 | 595.29883 | 2 | 44.6451 | 52800000 |
| PAIPAPPVGPGPK | 43.49 | 1196.6917 | 13 | 0.8 | 599.35358 | 2 | 27.7874 | 44900000 |
| AIPAPPVGPGPK | 43.39 | 1099.6389 | 12 | 0.9 | 550.82721 | 2 | 25.649 | 2020000 |
| GGFDFSFLPQPPQ | 43.17 | 1435.6772 | 13 | 1.3 | 718.8468 | 2 | 50.7454 | 16700000 |
| GFDFSFLPQPPQ | 42.57 | 1378.6557 | 12 | 0.6 | 690.33557 | 2 | 53.8779 | 43500000 |
| GDIGAAGAVGPSGPAGPR | 42.1 | 1505.7586 | 18 | 1.1 | 753.88739 | 2 | 21.4166 | 137000 |
| FDFSFLPQPPQE | 41.92 | 1450.6768 | 12 | 0.4 | 726.34601 | 2 | 51.432 | 60900000 |
| VDPAIPAPPVGPG | 41.7 | 1185.6393 | 13 | 0.5 | 593.82721 | 2 | 37.181 | 595000 |
| GEQGPSGASGPAGPR | 41.63 | 1323.6167 | 15 | 0.4 | 662.81592 | 2 | 9.7425 | 122000 |
| PAPPVGPGPK | 40.3 | 915.5178 | 10 | 0.1 | 458.7662 | 2 | 26.1239 | 277000 |
| GHRGFSGL | 40.28 | 829.4195 | 8 | 1.2 | 415.7175 | 2 | 15.5255 | 15300000 |
| SFLPQPPQE | 39.9 | 1041.5131 | 9 | 0.3 | 1042.52063 | 1 | 35.8601 | 51600000 |
| PAIPAPPVGPG | 39.75 | 971.544 | 11 | 1.3 | 486.77988 | 2 | 34.2284 | 5990000 |
| LGPQGAIGPA | 38.62 | 879.4814 | 10 | -0.1 | 880.48859 | 1 | 26.8154 | 1140000 |
| IPAPPVGPGPK | 38.36 | 1028.6018 | 11 | 1 | 515.30872 | 2 | 23.8255 | 9130000 |
| GPPGPPGPRGPSGSP | 38.18 | 1312.6523 | 15 | -15.6 | 657.32324 | 2 | 41.0875 |  |
| FDFSFLPQPPQ | 38.13 | 1321.6343 | 11 | 0.5 | 661.82471 | 2 | 52.2804 | 46800000 |
| GGFDFSFLPQPPQE | 38.08 | 1564.7198 | 14 | 1 | 783.36798 | 2 | 53.1785 | 12900000 |
| DFSFLPQPPQ | 38.06 | 1174.5658 | 10 | 0.7 | 588.29059 | 2 | 45.9358 | 265000000 |
| GFSGLDGAKGDTGPAGPK | 37.81 | 1630.795 | 18 | 4.2 | 544.60791 | 3 | 21.3837 | 156000 |
| SFLPQPPQ | 37.8 | 912.4705 | 8 | 1.2 | 913.47888 | 1 | 35.6121 | 11700000 |
| RGLTGPIGPPGPS | 37.76 | 1204.6564 | 13 | 0.5 | 603.33575 | 2 | 25.4634 | 6270000 |
| G(+42.01)PPGPPGAPSGGFDFSFLPQPPQE | 37.4 | 2421.1277 | 24 | 1 | 808.0506 | 3 | 52.256 |  |
| DNDIMLIKL | 37.29 | 1073.5791 | 9 | 1.4 | 537.79755 | 2 | 47.8919 | 1220000 |
| DNDLMLLKL | 37.29 | 1073.5791 | 9 | 1.4 | 537.79755 | 2 | 47.8919 | 1220000 |
| GFSGLDGAK | 37.23 | 850.4184 | 9 | 0.7 | 426.2168 | 2 | 19.3331 | 8010000 |
| GPAGPTGPH | 36.65 | 789.3769 | 9 | 2.5 | 395.69672 | 2 | 8.6929 | 232000 |
| VGPSGPAGPR | 36.57 | 893.4719 | 10 | 1.7 | 447.74396 | 2 | 10.8512 | 2450000 |
| SEPIRVLVTGAAGQIAY | 36.49 | 1743.9519 | 17 | -1 | 872.98236 | 2 | 53.4507 | 80800 |
| PGLIGPPGSP | 36.45 | 890.4861 | 10 | 0.9 | 891.4942 | 1 | 30.0334 | 205000 |
| TLDNDLMLLK | 36.29 | 1174.6267 | 10 | 0.8 | 588.32111 | 2 | 37.5332 | 1110000 |
| VDPAIPAPPVGPGPK | 35.85 | 1410.787 | 15 | 0.2 | 706.40094 | 2 | 31.7843 | 7540000 |
| PGPIGPEGPR | 35.61 | 975.5137 | 10 | 2.3 | 488.76526 | 2 | 23.8255 | 731000 |
| FSFLPQPPQ | 35.37 | 1059.5389 | 9 | 0.8 | 530.77716 | 2 | 44.7429 | 32000000 |
| GPMGPR | 35.28 | 613.3006 | 6 | 1.7 | 307.65811 | 2 | 8.9677 | 157000 |
| PSGGFDFSFLPQPPQ | 35.26 | 1619.762 | 15 | 0.7 | 810.88879 | 2 | 52.5042 | 296000 |
| G(+42.01)PPGAPSGGFDFSFLPQPPQE | 35.11 | 2170.0007 | 21 | 3 | 1086.01086 | 2 | 51.6088 | 281000 |
| DFSFLPQPP | 34.99 | 1046.5073 | 9 | -0.5 | 1047.51404 | 1 | 52.5202 | 8240000 |
| PAIPAPPVG | 34.86 | 817.4697 | 9 | 0.3 | 818.47723 | 1 | 33.3363 | 9220000 |
| G(+42.01)APGAPGPVGPAGKNGD | 34.34 | 1459.7055 | 17 | 1 | 730.86072 | 2 | 16.6629 |  |
| LPFPEGPL | 34.24 | 868.4694 | 8 | -0.1 | 869.47662 | 1 | 48.2907 | 104000 |
| PSGGFDFS | 34.16 | 812.3341 | 8 | 0.2 | 813.34149 | 1 | 31.1577 | 549000 |
| VDPAIPAPPVG | 34.11 | 1031.5651 | 11 | 0.1 | 516.78986 | 2 | 36.6088 | 6810000 |
| TGPTGPAGQDGRPGPA | 34.09 | 1434.6851 | 16 | -0.2 | 718.34967 | 2 | 12.5281 |  |
| AVGPSGPAGPR | 34.04 | 964.509 | 11 | 2.7 | 483.26306 | 2 | 11.3454 | 1360000 |
| VGPTGPA | 33.8 | 597.3122 | 7 | 1 | 598.32007 | 1 | 13.6805 | 1190000 |
| DFSFLPQPPQEK | 33.78 | 1431.7034 | 12 | 0.7 | 716.8595 | 2 | 39.1656 | 6160000 |
| IGPRGLPGE | 33.5 | 894.4923 | 9 | 0.8 | 448.25375 | 2 | 20.3395 | 468000 |
| GFSGLDGA | 33.47 | 722.3235 | 8 | 0.2 | 723.33093 | 1 | 28.8287 | 6750000 |
| P(+42.01)GAPSGGFDFSFLPQPPQ | 33.47 | 1886.8839 | 18 | -0.2 | 944.44897 | 2 | 52.3782 | 365000 |
| IGPRGLPGER | 33.34 | 1050.5934 | 10 | 1.2 | 351.20551 | 3 | 15.5255 | 911000 |
| PGPMGPAGPR | 33.13 | 935.4647 | 10 | -15.8 | 468.73224 | 2 | 21.8489 | 35800 |
| G(+42.01)APGKTGPVGPQ | 33.13 | 1106.572 | 12 | 17 | 554.30267 | 2 | 20.9288 |  |
| G(+42.01)QPGLSGVPGPM(+15.99) | 33.09 | 1153.5437 | 12 | 0.7 | 577.77954 | 2 | 25.1812 | 355000 |
| P(+42.01)GAPSGGFDFSFLPQPPQE | 33.06 | 2015.9265 | 19 | -0.8 | 1008.96973 | 2 | 52.1717 | 265000 |
| QPGLSGVPGPM | 32.93 | 1038.5168 | 11 | -0.7 | 1039.52332 | 1 | 43.3945 | 2510000 |
| GELGPQGAIGPA | 32.91 | 1065.5454 | 12 | 0.4 | 533.78021 | 2 | 28.8287 | 3710000 |
| GPMGIMGPR | 32.91 | 914.4466 | 9 | 1 | 458.23105 | 2 | 27.7242 | 666000 |
| HRGFSGL | 32.84 | 772.398 | 7 | 1.1 | 387.2067 | 2 | 18.0041 | 5380000 |
| FLPQPPQE | 32.54 | 954.4811 | 8 | -0.4 | 955.48798 | 1 | 39.9744 | 38000000 |
| GERGEQGSPGPSGFQ | 32.53 | 1488.6593 | 15 | 0.9 | 745.33759 | 2 | 14.6889 | 200000 |
| GLSGVPGPM(+15.99) | 32.09 | 829.4004 | 9 | 0.5 | 830.40808 | 1 | 24.5952 | 34100000 |
| GILTLKYPIE | 32.04 | 1145.6696 | 10 | 1 | 573.84265 | 2 | 41.0913 | 200000 |
| FSGLDGAK | 31.94 | 793.397 | 8 | 0.9 | 397.70615 | 2 | 18.2604 | 2550000 |
| SPGFDGLQGPPGPNGDL | 31.73 | 1623.7528 | 17 | 2.4 | 812.88562 | 2 | 36.3893 | 96200 |
| GFDFSFLPQ | 31.71 | 1056.4916 | 9 | -0.2 | 1057.49866 | 1 | 53.5494 | 742000 |
| PLGGHSGPA | 31.32 | 791.3926 | 9 | -14.1 | 792.38867 | 1 | 20.5388 | 958000 |
| GFDFSFL | 31.25 | 831.3803 | 7 | 0.2 | 832.3877 | 1 | 57.9625 | 367000 |
| GAVGPSGPAGPR | 31.05 | 1021.5304 | 12 | 2.4 | 511.77371 | 2 | 12.1682 | 1140000 |
| SFLPQPP | 30.94 | 784.4119 | 7 | -0.4 | 785.41888 | 1 | 39.2318 | 2650000 |
| AIPAPPVG | 30.94 | 720.417 | 8 | 0.1 | 721.42432 | 1 | 29.4841 | 2660000 |
| RSPMAP | 30.81 | 657.3268 | 6 | 9 | 658.34003 | 1 | 27.7871 | 1440000 |
| GPSGASGPAGPR | 30.8 | 1009.4941 | 12 | 3.6 | 505.75613 | 2 | 8.9586 | 21300 |
| VGPSGPAGPRG | 30.74 | 950.4933 | 11 | 3 | 476.25537 | 2 | 10.4335 | 13800 |
| A(+42.01)GPAGESGKP | 30.67 | 911.4348 | 10 | 0.6 | 456.72495 | 2 | 20.8816 | 77900 |
| TGPAGPAGPAGPA | 30.65 | 1019.5036 | 13 | 1.9 | 510.76004 | 2 | 18.2894 | 116000 |
| PMGIMGPR | 30.64 | 857.4251 | 8 | 0.4 | 429.72 | 2 | 28.7606 | 478000 |
| GPMGPAGPR | 30.62 | 838.4119 | 9 | 2.5 | 420.21429 | 2 | 14.1335 | 153000 |
| GPVGPAGL | 30.58 | 666.37 | 8 | 0.8 | 667.37787 | 1 | 28.8611 | 2780000 |
| PGAVGPVG | 30.22 | 652.3544 | 8 | 1 | 653.3623 | 1 | 20.1068 | 2460000 |
| GPAGPAGARGAP | 30.21 | 977.5042 | 12 | 2.7 | 489.76071 | 2 | 10.3094 |  |
| FDPSILFPK | 30.07 | 1062.5749 | 9 | 0.7 | 532.2951 | 2 | 42.2665 | 488000 |
| ASGPVGPA | 30.05 | 654.3337 | 8 | 1 | 655.34161 | 1 | 15.129 | 314000 |
| GLSGPPGPA | 30.05 | 751.3864 | 9 | 0.4 | 752.39398 | 1 | 19.717 | 11100000 |
| G(+42.01)PPGPPGAPSGGFDFSF | 29.91 | 1631.7256 | 17 | 0.4 | 816.87036 | 2 | 46.84 | 56800 |
| FDFSFLPQPPQEK | 29.9 | 1578.7718 | 13 | 0.2 | 790.39337 | 2 | 44.5802 | 957000 |
| GPPSPGVAPLA | 29.87 | 961.5232 | 11 | 0.8 | 481.76926 | 2 | 30.5025 | 202000 |
| GPAGPTGPHG | 29.82 | 846.3984 | 10 | 2.9 | 424.20767 | 2 | 8.7429 | 40200 |
| GHPGPIGPA | 29.81 | 801.4133 | 9 | 2 | 401.71472 | 2 | 15.3274 | 604000 |
| GPTGPIGPR | 29.76 | 850.4661 | 9 | 1 | 426.24072 | 2 | 16.473 | 57000 |
| G(+42.01)AGAPGAAGGGGGPGPGA | 29.75 | 1276.5796 | 18 | -18.8 | 639.28503 | 2 | 26.7096 |  |
| K(+42.01)GVPGDKGPR | 29.64 | 1051.5774 | 10 | -0.6 | 351.53287 | 3 | 15.2771 |  |
| DFSFLPQ | 29.63 | 852.4017 | 7 | 0.1 | 853.40912 | 1 | 48.4895 | 3370000 |
| GVPGVPGF | 29.6 | 728.3857 | 8 | 0.9 | 729.39362 | 1 | 36.4788 | 894000 |
| GLSGVPGPM | 29.6 | 813.4054 | 9 | 0.4 | 407.71017 | 2 | 33.78 | 96100000 |
| G(+42.01)PPGAPSGGFDFSF | 29.52 | 1380.5986 | 14 | 0.5 | 691.30688 | 2 | 45.3435 | 1350000 |
| FGGL | 29.35 | 392.2059 | 4 | 1.1 | 393.21365 | 1 | 26.8506 | 1140000 |
| FGGI | 29.35 | 392.2059 | 4 | 1.1 | 393.21365 | 1 | 26.8506 | 1140000 |
| Q(+42.01)PAPIGTQGLH | 29.23 | 1159.5985 | 11 | 0.7 | 580.80695 | 2 | 20.1068 | 1430000 |
| GAVGPVGPR | 29.17 | 808.4555 | 9 | 1.7 | 405.23572 | 2 | 14.6148 |  |
| GLAGPQGPR | 29.15 | 851.4613 | 9 | 2 | 426.7388 | 2 | 14.0654 | 79900 |
| GLPGPPGSPGL | 29.07 | 947.5076 | 11 | 0.8 | 474.76144 | 2 | 34.014 | 2380000 |
| PGSPGPPGPRGYPG | 29.03 | 1291.6309 | 14 | 19.3 | 646.83521 | 2 | 23.2588 |  |
| TGDVGPA | 29 | 615.2864 | 7 | 1.2 | 616.29437 | 1 | 12.1395 | 210000 |
| SGPAGPVG | 28.95 | 640.318 | 8 | 1.3 | 641.32611 | 1 | 13.9817 | 247000 |
| GERGFPGL | 28.93 | 831.4239 | 8 | 0.7 | 416.71948 | 2 | 29.1556 | 314000 |
| PAIPAPPVGPGP | 28.92 | 1068.5967 | 12 | -0.4 | 535.30542 | 2 | 35.909 | 349000 |
| FPLP | 28.84 | 472.2685 | 4 | 0.4 | 473.27603 | 1 | 39.8024 | 914000 |
| FPIP | 28.84 | 472.2685 | 4 | 0.4 | 473.27603 | 1 | 39.8024 | 914000 |
| IPAPPVGPG | 28.83 | 803.4541 | 9 | 0.7 | 402.73462 | 2 | 28.6069 | 483000 |
| IGGF | 28.8 | 392.2059 | 4 | 0.6 | 393.21347 | 1 | 29.3853 | 1620000 |
| LGGF | 28.8 | 392.2059 | 4 | 0.6 | 393.21347 | 1 | 29.3853 | 1620000 |
| LPGPPGSP | 28.65 | 720.3806 | 8 | 0.8 | 721.38849 | 1 | 22.7675 | 2910000 |
| SVGGGL | 28.63 | 488.2594 | 6 | 1 | 489.26721 | 1 | 19.0967 | 277000 |
| GPSGPSGPRGP | 28.59 | 964.4726 | 11 | -12.7 | 483.23746 | 2 | 16.3394 |  |
| PGPMGPRGAP | 28.56 | 935.4647 | 10 | 8.3 | 468.7435 | 2 | 22.4639 |  |
| PGER | 28.53 | 457.2285 | 4 | 1.4 | 458.23639 | 1 | 11.1124 | 80500 |
| SFGL | 28.52 | 422.2165 | 4 | 0.6 | 423.22406 | 1 | 32.1309 | 652000 |
| SFGI | 28.52 | 422.2165 | 4 | 0.6 | 423.22406 | 1 | 32.1309 | 652000 |
| LTGPIGPPGPA | 28.5 | 975.5389 | 11 | -0.2 | 488.77664 | 2 | 39.0043 | 429000 |
| GGFDFSF | 28.41 | 775.3177 | 7 | 0.5 | 776.32532 | 1 | 47.8095 | 6790000 |
| FDFSFL | 28.4 | 774.3588 | 6 | 0.5 | 775.36646 | 1 | 55.7984 | 1160000 |
| GPMGIM | 28.35 | 604.2713 | 6 | 0.9 | 605.27911 | 1 | 36.1227 | 6570000 |
| R(+42.01)GPAGPI | 28.34 | 708.3918 | 7 | -15.1 | 709.38843 | 1 | 23.5316 | 373000 |
| GFDF | 28.33 | 484.1958 | 4 | 0.8 | 485.20343 | 1 | 33.6194 | 795000 |
| GPPGLAGPPGSP | 28.27 | 1002.5134 | 12 | 0 | 1003.52069 | 1 | 24.0406 | 126000 |
| A(+42.01)GPIGPPGER | 28.26 | 991.5087 | 10 | 0.8 | 496.76202 | 2 | 20.1374 | 15900000 |
| GPVGERGAPGNRGF | 28.22 | 1369.685 | 14 | -7.1 | 685.84497 | 2 | 13.1622 | 55900 |
| LPF | 28.19 | 375.2158 | 3 | 0.9 | 376.22342 | 1 | 33.1478 | 925000 |
| IPF | 28.19 | 375.2158 | 3 | 0.9 | 376.22342 | 1 | 33.1478 | 925000 |
| Q(+42.01)VAVAAGSLPSPAGP | 28.18 | 1362.7143 | 15 | 7.5 | 682.36951 | 2 | 27.4419 | 374000 |
| GPPGLPGAP | 28.13 | 761.4071 | 9 | 0.7 | 381.71112 | 2 | 24.4795 | 217000 |
| WEPAPGPV | 28.06 | 851.4177 | 8 | 1 | 426.71655 | 2 | 31.5 | 659000 |
| SGPVGPAGPAGARGAP | 27.92 | 1317.6789 | 16 | 1.2 | 659.84753 | 2 | 18.8001 | 133000 |
| K(+42.01)PSVSLY | 27.9 | 834.4487 | 7 | 0.3 | 835.45624 | 1 | 33.2377 | 1960000 |
| GPRPSESIVVPA | 27.89 | 1207.656 | 12 | 0.9 | 604.83582 | 2 | 25.919 | 343000 |
| FSGLDGA | 27.84 | 665.302 | 7 | -0.1 | 666.30927 | 1 | 25.4318 | 2120000 |
| GSDGISGPK | 27.84 | 816.3977 | 9 | 2.6 | 409.20721 | 2 | 9.2506 |  |
| FLAPGAPDAPA | 27.82 | 1025.5181 | 11 | 0.6 | 1026.526 | 1 | 37.4761 | 2470000 |
| FLPQPPQ | 27.8 | 825.4385 | 7 | 0.2 | 826.44592 | 1 | 39.8024 | 7340000 |
| VFPSIVG | 27.68 | 717.4061 | 7 | 0.3 | 718.41357 | 1 | 37.9755 | 7190000 |
| GPM(+15.99)GIM(+15.99)GPR | 27.64 | 946.4364 | 9 | 1 | 474.22595 | 2 | 13.1692 |  |
| LGPA | 27.62 | 356.2059 | 4 | 2.1 | 357.21396 | 1 | 12.6993 | 697000 |
| IGPA | 27.62 | 356.2059 | 4 | 2.1 | 357.21396 | 1 | 12.6993 | 697000 |
| A(+42.01)GPIGPPGPA | 27.58 | 874.4548 | 10 | 5.6 | 438.23712 | 2 | 21.471 | 148000 |
| LGYT | 27.55 | 452.2271 | 4 | -14.3 | 453.22791 | 1 | 19.9082 | 278000 |
| IGYT | 27.55 | 452.2271 | 4 | -14.3 | 453.22791 | 1 | 19.9082 | 278000 |
| PGGAGPIGPPGER | 27.5 | 1160.5938 | 13 | 6.1 | 581.30768 | 2 | 20.0577 |  |
| VGPRPSESIVVPA | 27.5 | 1306.7245 | 13 | 0.7 | 654.37 | 2 | 28.7952 | 213000 |
| PGVGGVPG | 27.49 | 638.3387 | 8 | 1 | 639.34668 | 1 | 19.2314 | 1020000 |
| GQIGPRGLPGER | 27.42 | 1235.6734 | 12 | 0.7 | 412.89871 | 3 | 16.9161 | 97300 |
| LLGL | 27.41 | 414.2842 | 4 | 1.1 | 415.29193 | 1 | 45.3661 | 3770000 |
| IIGL | 27.41 | 414.2842 | 4 | 1.1 | 415.29193 | 1 | 45.3661 | 3770000 |
| LLGI | 27.41 | 414.2842 | 4 | 1.1 | 415.29193 | 1 | 45.3661 | 3770000 |
| LIGL | 27.41 | 414.2842 | 4 | 1.1 | 415.29193 | 1 | 45.3661 | 3770000 |
| ILGL | 27.41 | 414.2842 | 4 | 1.1 | 415.29193 | 1 | 45.3661 | 3770000 |
| LIGI | 27.41 | 414.2842 | 4 | 1.1 | 415.29193 | 1 | 45.3661 | 3770000 |
| ILGI | 27.41 | 414.2842 | 4 | 1.1 | 415.29193 | 1 | 45.3661 | 3770000 |
| GPNGDPGPLG | 27.4 | 879.4086 | 10 | 5.5 | 440.71399 | 2 | 38.4815 |  |
| R(+42.01)PGPPGSP | 27.38 | 805.4082 | 8 | -15.7 | 806.40283 | 1 | 28.574 | 144000 |
| GPLGGF | 27.37 | 546.2802 | 6 | 0.7 | 547.28784 | 1 | 30.5305 | 1050000 |
| GFDFSF | 27.37 | 718.2962 | 6 | 0.4 | 719.30377 | 1 | 45.385 | 19900000 |
| LGGL | 27.32 | 358.2216 | 4 | 1 | 359.22925 | 1 | 27.2576 | 1080000 |
| IGGL | 27.32 | 358.2216 | 4 | 1 | 359.22925 | 1 | 27.2576 | 1080000 |
| LGGI | 27.32 | 358.2216 | 4 | 1 | 359.22925 | 1 | 27.2576 | 1080000 |
| IGGI | 27.32 | 358.2216 | 4 | 1 | 359.22925 | 1 | 27.2576 | 1080000 |
| GPAGPIGDPGKEGP | 27.29 | 1247.6146 | 14 | 9.8 | 624.82068 | 2 | 17.4061 |  |
| LPPW | 27.22 | 511.2794 | 4 | 1.1 | 512.28729 | 1 | 35.9635 | 1110000 |
| IPPW | 27.22 | 511.2794 | 4 | 1.1 | 512.28729 | 1 | 35.9635 | 1110000 |
| GAPGERGPAGL | 27.21 | 980.5039 | 11 | 1.8 | 491.2601 | 2 | 16.6458 | 96400 |
| PGAWPAP | 27.2 | 694.3438 | 7 | 1 | 695.35181 | 1 | 43.2695 | 2000000 |
| PIGPEGPR | 27.2 | 821.4395 | 8 | 0.8 | 411.72736 | 2 | 24.5865 | 1200000 |
| FDFSF | 27.19 | 661.2748 | 5 | 0 | 662.28204 | 1 | 45.9557 | 45700000 |
| G(+42.01)PPGAPSGGFDFSFLPQPPQ | 27.13 | 2040.9581 | 20 | -0.6 | 1021.48572 | 2 | 51.9452 | 219000 |
| FPGL | 27.12 | 432.2372 | 4 | 0.6 | 433.24478 | 1 | 34.8628 | 1610000 |
| YPIE | 27.12 | 520.2533 | 4 | -2 | 521.25952 | 1 | 19.7968 | 161000 |
| YPLE | 27.12 | 520.2533 | 4 | -2 | 521.25952 | 1 | 19.7968 | 161000 |
| FPGI | 27.12 | 432.2372 | 4 | 0.6 | 433.24478 | 1 | 34.8628 | 1610000 |
| N(+42.01)APPAGLSPAPGGAP | 27.1 | 1314.6568 | 15 | 9.5 | 658.34192 | 2 | 17.5417 |  |
| RGFSGLDGA | 27.04 | 878.4246 | 9 | -0.2 | 440.21948 | 2 | 24.0735 | 135000 |
| Q(+42.01)LGFSGPA | 26.99 | 817.397 | 8 | -0.1 | 818.40417 | 1 | 30.7943 | 4850000 |
| IPVV | 26.97 | 426.2842 | 4 | 1.1 | 427.29196 | 1 | 28.3289 | 2490000 |
| LPVV | 26.97 | 426.2842 | 4 | 1.1 | 427.29196 | 1 | 28.3289 | 2490000 |
| GPEGAPGPVGPA | 26.96 | 1004.4927 | 12 | 0.4 | 503.25381 | 2 | 17.3969 | 279000 |
| DFSFLPQP | 26.94 | 949.4545 | 8 | 0.1 | 950.46185 | 1 | 50.983 | 67100 |
| TPGAPGLP | 26.87 | 708.3806 | 8 | 0.8 | 709.38843 | 1 | 23.5316 | 373000 |
| GFSGL | 26.83 | 479.238 | 5 | 0.7 | 480.24561 | 1 | 28.0751 | 20800000 |
| GFSGI | 26.83 | 479.238 | 5 | 0.7 | 480.24561 | 1 | 28.0751 | 20800000 |
| PGPIGPA | 26.78 | 607.3329 | 7 | 0.7 | 608.34064 | 1 | 20.5664 | 252000 |
| G(+42.01)APGSTGPAGPRGPA | 26.75 | 1290.6316 | 15 | 0 | 646.32306 | 2 | 24.3651 | 47000 |
| FSFI | 26.73 | 512.2635 | 4 | 0.6 | 513.27106 | 1 | 42.974 | 715000 |
| FSFL | 26.73 | 512.2635 | 4 | 0.6 | 513.27106 | 1 | 42.974 | 715000 |
| IRHFQV | 26.73 | 798.45 | 6 | 1.2 | 400.23276 | 2 | 18.0823 | 2920000 |
| TGPIGPPGPA | 26.67 | 862.4548 | 10 | 0.4 | 432.23486 | 2 | 25.3984 | 184000 |
| GDTGPVG | 26.62 | 601.2707 | 7 | 2.4 | 602.27942 | 1 | 11.5576 | 102000 |
| PGLPFHP | 26.54 | 763.4017 | 7 | 0.1 | 764.409 | 1 | 32.8369 | 10200000 |
