# Supplementary material for: Tortoise Oligopeptides Augment Cyclophosphamide's Antitumor Activity Through Dual Modulation of Therapeutic Efficacy and Hematologic Toxicity
Source: Food Sci Nutr. 2025 Oct 16;13(10):e71078. doi: 10.1002/fsn3.71078 (PMC12531418; doi:10.1002/fsn3.71078)
Supplement: Supplementary file 3 — Data S3: fsn371078‐sup‐0003‐Supplement3.docx. [file FSN3-13-e71078-s003.docx]

| **Peptide** | **bioactivity** | **Screening** | **-10lgP** | **Mass** | **Length** | **ppm** | **m/z** | **z** | **RT** | **Area Sample 1** |
| --- | --- | --- | --- | --- | --- | --- | --- | --- | --- | --- |
| LPGPF | 0.972673 | 0.972673 | 20.3 | 529.29 | 5 | 0.8 | 530.29773 | 1 | 41.41 | 80100000 |
| IPGPF | 0.965292 | 0.965292 | 20.3 | 529.29 | 5 | 0.8 | 530.29773 | 1 | 41.41 | 80100000 |
| PAIPAPPVGPGPK | 0.826454 | 0.826454 | 43.49 | 1196.6917 | 13 | 0.8 | 599.35358 | 2 | 27.7874 | 44900000 |
| FSFPTLPF | 0.9449 | 0.9449 | 8.6 | 954.4851 | 8 | -3.6 | 478.24811 | 2 | 39.9765 | 26300000 |
| PGPMGPRGPA | 0.806208 | 0.806208 | 11.22 | 935.4647 | 10 | 8 | 468.74338 | 2 | 21.3237 | 22200000 |
| GPVGPSGPPGIP | 0.801538 | 0.801538 | 15.69 | 1030.5447 | 12 | 0.3 | 516.27979 | 2 | 35.0932 | 12600000 |
| GLPFHP | 0.889786 | 0.889786 | 18.52 | 666.3489 | 6 | 0.3 | 334.18182 | 2 | 25.3722 | 11100000 |
| GPYGL | 0.876044 | 0.876044 | 11.02 | 505.2536 | 5 | 1 | 506.26141 | 1 | 31.9098 | 10900000 |
| SGPPGLPGPIGLPGDPG | 0.925796 | 0.925796 | 7.76 | 1483.767 | 17 | 5.4 | 495.59897 | 3 | 20.0476 | 10400000 |
| PGLPFHP | 0.877478 | 0.877478 | 26.54 | 763.4017 | 7 | 0.1 | 764.409 | 1 | 32.8369 | 10200000 |
| PPSPL | 0.856544 | 0.856544 | 14.38 | 509.2849 | 5 | 0.7 | 510.29254 | 1 | 46.3363 | 9590000 |
| GPPGPNGNPGPPGPPGA | 0.877299 | 0.877299 | 6.76 | 1435.6844 | 17 | -4.1 | 718.8465 | 2 | 53.5105 | 9130000 |
| DFSFLPQPP | 0.823224 | 0.823224 | 34.99 | 1046.5073 | 9 | -0.5 | 1047.51404 | 1 | 52.5202 | 8240000 |
| GSPGFPGS | 0.811979 | 0.811979 | 22.89 | 704.3129 | 8 | 0.8 | 705.32074 | 1 | 19.1759 | 8220000 |
| GLLGF | 0.905756 | 0.905756 | 21.25 | 505.29 | 5 | 1.1 | 506.29785 | 1 | 45.4059 | 8190000 |
| GILGF | 0.867055 | 0.867055 | 21.25 | 505.29 | 5 | 1.1 | 506.29785 | 1 | 45.4059 | 8190000 |
| LGLF | 0.919409 | 0.919409 | 24.09 | 448.2685 | 4 | 0.9 | 449.27625 | 1 | 42.4617 | 8100000 |
| PGLSGPPGPVGP | 0.813917 | 0.813917 | 15.44 | 1030.5447 | 12 | 1.5 | 516.2804 | 2 | 52.3032 | 8090000 |
| GFPGLPGSP | 0.840122 | 0.840122 | 14.01 | 827.4177 | 9 | 0 | 828.42499 | 1 | 36.2905 | 7840000 |
| SPPPI | 0.828015 | 0.828015 | 14.13 | 509.2849 | 5 | 0.7 | 510.29254 | 1 | 46.3363 | 7670000 |
| SPPPL | 0.911525 | 0.911525 | 14.13 | 509.2849 | 5 | 0.7 | 510.29254 | 1 | 46.3363 | 7670000 |
| GGFDFSF | 0.949751 | 0.949751 | 28.41 | 775.3177 | 7 | 0.5 | 776.32532 | 1 | 47.8095 | 6790000 |
| LPGFPPH | 0.891033 | 0.891033 | 11.53 | 763.4017 | 7 | 0.7 | 382.70837 | 2 | 28.7639 | 6510000 |
| QPGLDGQPGPPGPPGARG | 0.863172 | 0.863172 | 6.89 | 1653.8223 | 18 | 15.9 | 827.93152 | 2 | 31.2842 | 5520000 |
| FSFLP | 0.968869 | 0.968869 | 20.61 | 609.3162 | 5 | 0.9 | 610.32404 | 1 | 45.8589 | 4880000 |
| GPIDEPPPPL | 0.814034 | 0.814034 | 10.51 | 1030.5335 | 10 | 12.5 | 516.28046 | 2 | 49.4259 | 4780000 |
| GPGEPPPPPPQ | 0.800505 | 0.800505 | 13.03 | 1068.524 | 11 | 1.6 | 535.27014 | 2 | 18.3558 | 4570000 |
| GPPGPTGALGPA | 0.814778 | 0.814778 | 9.65 | 990.5134 | 12 | 0.1 | 496.26404 | 2 | 39.5419 | 4100000 |
| QGPAPPTGPRPT | 0.810765 | 0.810765 | 5.52 | 1174.6094 | 12 | 0.8 | 588.31244 | 2 | 18.978 | 3920000 |
| IPGRGLPGFPG | 0.805927 | 0.805927 | 12.88 | 1066.5923 | 11 | -1.5 | 356.53751 | 3 | 12.5626 | 3880000 |
| FSTLRFFEGF | 0.850184 | 0.850184 | 14.55 | 1249.6131 | 10 | -3.1 | 625.81189 | 2 | 33.747 | 3780000 |
| GLSGPPGLPGPIGLPGDPG | 0.910212 | 0.910212 | 8.14 | 1653.8725 | 19 | -15.4 | 827.93085 | 2 | 32.2836 | 3560000 |
| GLGPF | 0.959912 | 0.959912 | 13.18 | 489.2587 | 5 | 1 | 490.26648 | 1 | 36.7564 | 3380000 |
| GVPGPM | 0.819821 | 0.819821 | 21.52 | 556.2679 | 6 | 0.6 | 557.27551 | 1 | 22.4254 | 3240000 |
| DRFPGPA | 0.812689 | 0.812689 | 8.33 | 758.3711 | 7 | -14.9 | 759.36707 | 1 | 36.226 | 3060000 |
| PGYGLP | 0.850915 | 0.850915 | 19.14 | 602.3064 | 6 | 0.6 | 603.31403 | 1 | 36.5057 | 2890000 |
| GPIGYPG | 0.833133 | 0.833133 | 14.01 | 659.3278 | 7 | -0.1 | 660.33508 | 1 | 33.1394 | 2670000 |
| GPLGYPG | 0.835911 | 0.835911 | 14.01 | 659.3278 | 7 | -0.1 | 660.33508 | 1 | 33.1394 | 2670000 |
| GYPGIGKPGMP | 0.838496 | 0.838496 | 5.04 | 1072.5375 | 11 | -6.1 | 537.27277 | 2 | 24.7318 | 2660000 |
| SFLPQPP | 0.850466 | 0.850466 | 30.94 | 784.4119 | 7 | -0.4 | 785.41888 | 1 | 39.2318 | 2650000 |
| SPGAGGGGGGFF | 0.949994 | 0.949994 | 17.26 | 966.4195 | 12 | 7 | 484.2204 | 2 | 32.1613 | 2470000 |
| GLPGPPGSPGL | 0.886809 | 0.886809 | 29.07 | 947.5076 | 11 | 0.8 | 474.76144 | 2 | 34.014 | 2380000 |
| GPSFR | 0.890458 | 0.890458 | 12.88 | 562.2863 | 5 | -19.3 | 563.28278 | 1 | 22.4254 | 2350000 |
| PGEPGPSGPPGNMGP | 0.916336 | 0.916336 | 6.29 | 1346.5925 | 15 | -10.7 | 674.29633 | 2 | 33.1478 | 2320000 |
| PGPPGPPGVPGLP | 0.938449 | 0.938449 | 13.58 | 1137.6182 | 13 | -13.3 | 569.80878 | 2 | 36.6088 | 2200000 |
| FLGGL | 0.913573 | 0.913573 | 19.34 | 505.29 | 5 | 1.2 | 506.29791 | 1 | 47.7919 | 2130000 |
| FLGGI | 0.818353 | 0.818353 | 19.34 | 505.29 | 5 | 1.2 | 506.29791 | 1 | 47.7919 | 2130000 |
| SPGPGAAMPPPP | 0.8339 | 0.8339 | 7.6 | 1074.5168 | 12 | -6.8 | 538.26202 | 2 | 24.5788 | 2120000 |
| FDFSFLPQPP | 0.823994 | 0.823994 | 25.68 | 1193.5757 | 10 | 0.4 | 1194.58337 | 1 | 55.1526 | 2110000 |
| GIIGLPGFPG | 0.821873 | 0.821873 | 14.83 | 926.5225 | 10 | -4.1 | 464.26663 | 2 | 28.9627 | 2030000 |
| PGSGPGSGAGSMRGAP | 0.827469 | 0.827469 | 7.81 | 1341.6095 | 16 | -5.8 | 671.80817 | 2 | 25.0133 | 1950000 |
| FLPQPP | 0.896473 | 0.896473 | 25.35 | 697.3799 | 6 | 0.8 | 698.3877 | 1 | 43.902 | 1920000 |
| PYDFPF | 0.98041 | 0.98041 | 20.96 | 784.3432 | 6 | 0.5 | 785.35083 | 1 | 50.1294 | 1870000 |
| GPQGLPGL | 0.856312 | 0.856312 | 17.58 | 737.4072 | 8 | 0.4 | 738.41473 | 1 | 33.3666 | 1790000 |
| GPQGIPGL | 0.853711 | 0.853711 | 17.58 | 737.4072 | 8 | 0.4 | 738.41473 | 1 | 33.3666 | 1790000 |
| GPYGGLL | 0.906144 | 0.906144 | 11.4 | 675.3591 | 7 | -0.2 | 676.36627 | 1 | 38.6655 | 1780000 |
| GPPGEPGPRGPPGAP | 0.88521 | 0.88521 | 8.27 | 1338.668 | 15 | -4.2 | 670.33844 | 2 | 51.6947 | 1740000 |
| PGSPF | 0.938334 | 0.938334 | 15.87 | 503.238 | 5 | 1.1 | 504.24582 | 1 | 47.8095 | 1730000 |
| PGSGGKAGGRGGPGG | 0.846288 | 0.846288 | 13.52 | 1167.5744 | 15 | 15.7 | 584.80365 | 2 | 24.6202 | 1700000 |
| QPGGMSPGMWPA | 0.857962 | 0.857962 | 6.23 | 1214.5212 | 12 | 5.1 | 608.271 | 2 | 20.1068 | 1650000 |
| FPGL | 0.972542 | 0.972542 | 27.12 | 432.2372 | 4 | 0.6 | 433.24478 | 1 | 34.8628 | 1610000 |
| FPGI | 0.937903 | 0.937903 | 27.12 | 432.2372 | 4 | 0.6 | 433.24478 | 1 | 34.8628 | 1610000 |
| HPFDLPA | 0.802705 | 0.802705 | 11.89 | 795.3915 | 7 | -13.7 | 796.38788 | 1 | 51.8891 | 1610000 |
| SGPPGPQGPPGA | 0.839851 | 0.839851 | 6.66 | 1017.4879 | 12 | 1.2 | 509.75186 | 2 | 19.5716 | 1550000 |
| TSWPHVGYRW | 0.806665 | 0.806665 | 7.35 | 1287.6149 | 10 | -2.2 | 644.81329 | 2 | 23.0556 | 1540000 |
| GPTGGFGF | 0.903231 | 0.903231 | 17.25 | 738.3337 | 8 | 0.2 | 739.34106 | 1 | 32.4132 | 1490000 |
| DFFSFA | 0.918511 | 0.918511 | 20.87 | 732.3119 | 6 | 0.3 | 733.3194 | 1 | 43.7166 | 1460000 |
| GIPGGL | 0.832835 | 0.832835 | 22.58 | 512.2958 | 6 | 1.1 | 513.30365 | 1 | 28.5735 | 1420000 |
| GPPGFPGAP | 0.9399 | 0.9399 | 10.26 | 795.3915 | 9 | 5.1 | 796.40283 | 1 | 53.0816 | 1400000 |
| PGIPGF | 0.93697 | 0.93697 | 19.36 | 586.3115 | 6 | 1.2 | 587.31946 | 1 | 41.0031 | 1380000 |
| PGLPGF | 0.948516 | 0.948516 | 19.36 | 586.3115 | 6 | 1.2 | 587.31946 | 1 | 41.0031 | 1380000 |
| LGGGFL | 0.898187 | 0.898187 | 11.02 | 562.3115 | 6 | 1.2 | 563.3194 | 1 | 42.2391 | 1380000 |
| PGPPGPPGVPGLPGEP | 0.913446 | 0.913446 | 11.92 | 1420.735 | 16 | -11.9 | 711.36633 | 2 | 31.9263 | 1360000 |
| PSPPL | 0.872994 | 0.872994 | 14.77 | 509.2849 | 5 | 1.4 | 510.29294 | 1 | 44.6502 | 1330000 |
| PGYPGL | 0.881417 | 0.881417 | 16.63 | 602.3064 | 6 | 0.6 | 603.31403 | 1 | 36.5057 | 1300000 |
| RWWPSGP | 0.928782 | 0.928782 | 14.88 | 884.4293 | 7 | 11 | 885.44629 | 1 | 36.0944 | 1280000 |
| AHGFL | 0.890533 | 0.890533 | 24.56 | 543.2805 | 5 | -19.2 | 544.27734 | 1 | 49.1869 | 1270000 |
| PGSPGAPGLIGNPGA | 0.844105 | 0.844105 | 6.61 | 1260.6462 | 15 | 9.2 | 631.33618 | 2 | 18.392 | 1260000 |
| EGSPGFGL | 0.82723 | 0.82723 | 13.24 | 762.3548 | 8 | -1.2 | 763.36115 | 1 | 31.813 | 1230000 |
| GFGPGLA | 0.825786 | 0.825786 | 21.85 | 617.3173 | 7 | 1.3 | 618.32538 | 1 | 33.2705 | 1210000 |
| GFPGVGPGGGG | 0.86892 | 0.86892 | 13.68 | 857.4031 | 11 | 8.6 | 858.41779 | 1 | 41.599 | 1210000 |
| PGYPGP | 0.883162 | 0.883162 | 17.44 | 586.2751 | 6 | 0.8 | 587.28284 | 1 | 25.7935 | 1190000 |
| LPSPPW | 0.939256 | 0.939256 | 15.89 | 695.3642 | 6 | 1.5 | 696.37256 | 1 | 41.1948 | 1160000 |
| SPLPPW | 0.96338 | 0.96338 | 15.74 | 695.3642 | 6 | 1.5 | 696.37256 | 1 | 41.1948 | 1160000 |
| FPHLP | 0.924141 | 0.924141 | 13.2 | 609.3275 | 5 | 0.6 | 305.67117 | 2 | 26.8506 | 1150000 |
| PSPGAMLGPSPG | 0.802564 | 0.802564 | 7.2 | 1066.5117 | 12 | 17 | 534.27222 | 2 | 32.5028 | 1150000 |
| RPPFLSF | 0.939862 | 0.939862 | 5.24 | 862.4701 | 7 | 0.7 | 432.24261 | 2 | 47.4994 | 1110000 |
| GPRGDGGPPGL | 0.87352 | 0.87352 | 5.32 | 978.4882 | 11 | -10.5 | 490.24625 | 2 | 36.8226 | 1090000 |
| GPAGPNGFAGPPGA | 0.890736 | 0.890736 | 9.39 | 1165.5516 | 14 | 9.8 | 583.78876 | 2 | 27.417 | 1080000 |
| GPLGGF | 0.936266 | 0.936266 | 27.37 | 546.2802 | 6 | 0.7 | 547.28784 | 1 | 30.5305 | 1050000 |
| FPQGSDLAPGGGGG | 0.835166 | 0.835166 | 7.22 | 1215.552 | 14 | 16 | 608.79297 | 2 | 31.0597 | 1040000 |
| GPPGPNGPQGPPGPP | 0.817285 | 0.817285 | 24.94 | 1321.6414 | 15 | -7.5 | 661.82306 | 2 | 22.6694 | 1010000 |
| GLPGF | 0.960416 | 0.960416 | 19.87 | 489.2587 | 5 | 1.2 | 490.26657 | 1 | 34.5537 | 985000 |
| FPGGL | 0.966364 | 0.966364 | 18.32 | 489.2587 | 5 | 1.2 | 490.26657 | 1 | 34.5537 | 985000 |
| FPGGI | 0.929142 | 0.929142 | 18.32 | 489.2587 | 5 | 1.2 | 490.26657 | 1 | 34.5537 | 985000 |
| SPGFPGS | 0.812598 | 0.812598 | 16.89 | 647.2915 | 7 | 0.8 | 648.29926 | 1 | 18.4151 | 979000 |
| GPPGSPGPPGLDGLPG | 0.938295 | 0.938295 | 10.9 | 1370.683 | 16 | 9 | 686.35492 | 2 | 19.7136 | 952000 |
| FGVGPL | 0.813376 | 0.813376 | 16.57 | 588.3271 | 6 | 0.5 | 589.33472 | 1 | 39.8687 | 927000 |
| GPQGFL | 0.945693 | 0.945693 | 23.15 | 617.3173 | 6 | 0.2 | 618.32471 | 1 | 31.7843 | 926000 |
| QPLGGF | 0.898867 | 0.898867 | 18.25 | 617.3173 | 6 | 0.2 | 618.32471 | 1 | 31.7843 | 926000 |
| LGPQGF | 0.828322 | 0.828322 | 16.1 | 617.3173 | 6 | 0.2 | 618.32471 | 1 | 31.7843 | 926000 |
| PFFP | 0.993033 | 0.993033 | 14.62 | 506.2529 | 4 | 7.9 | 507.26419 | 1 | 49.9611 | 918000 |
| GPSGPPGAPGAPG | 0.831826 | 0.831826 | 8.76 | 1017.4879 | 13 | -9.7 | 509.74631 | 2 | 26.5616 | 908000 |
| GIFGL | 0.901007 | 0.901007 | 13.38 | 505.29 | 5 | 1.7 | 506.29813 | 1 | 46.1729 | 894000 |
| GLFGL | 0.908448 | 0.908448 | 13.38 | 505.29 | 5 | 1.7 | 506.29813 | 1 | 46.1729 | 894000 |
| PGSPGFPGL | 0.960517 | 0.960517 | 14.28 | 827.4177 | 9 | 0.5 | 828.42542 | 1 | 39.966 | 889000 |
| GFPGF | 0.990655 | 0.990655 | 16.36 | 523.2431 | 5 | 5.3 | 524.25311 | 1 | 39.7065 | 860000 |
| WTPYGP | 0.877817 | 0.877817 | 8.04 | 719.3279 | 6 | 3.7 | 720.33783 | 1 | 39.0043 | 854000 |
| GPPGPSGPPGVT | 0.836799 | 0.836799 | 10.68 | 1018.5083 | 12 | 0.6 | 510.26172 | 2 | 22.2333 | 814000 |
| GFLGAL | 0.822108 | 0.822108 | 14.25 | 576.3271 | 6 | 0.1 | 577.33447 | 1 | 44.5089 | 796000 |
| PPAPPPPPQ | 0.916165 | 0.916165 | 10.12 | 896.4756 | 9 | 1.1 | 449.24557 | 2 | 37.2789 | 796000 |
| LMPANPWGAP | 0.83862 | 0.83862 | 7.79 | 1052.5113 | 10 | 15.7 | 527.27118 | 2 | 52.3469 | 790000 |
| GPPGPPGPPGLGGNF | 0.982624 | 0.982624 | 7.98 | 1316.6513 | 15 | 15.5 | 659.34314 | 2 | 37.3654 | 789000 |
| GPVGPPGDRGFT | 0.858887 | 0.858887 | 20.27 | 1155.5672 | 12 | 1.2 | 578.79156 | 2 | 21.4436 | 777000 |
| GPAGPVSPPGNPGPA | 0.854696 | 0.854696 | 8.47 | 1270.6305 | 15 | 15.2 | 636.33221 | 2 | 41.9649 | 760000 |
| SPGPPRPLPV | 0.861546 | 0.861546 | 7.69 | 1015.5814 | 10 | 0.6 | 508.79828 | 2 | 37.2481 | 759000 |
| LGPGL | 0.805552 | 0.805552 | 21.9 | 455.2744 | 5 | 1 | 456.28207 | 1 | 33.5596 | 756000 |
| FLGGF | 0.979407 | 0.979407 | 18.46 | 539.2744 | 5 | 1 | 540.28217 | 1 | 49.8904 | 750000 |
| PGPM | 0.954011 | 0.954011 | 24.55 | 400.178 | 4 | 0.8 | 401.18561 | 1 | 22.4291 | 746000 |
| GFDFSFLPQ | 0.813978 | 0.813978 | 31.71 | 1056.4916 | 9 | -0.2 | 1057.49866 | 1 | 53.5494 | 742000 |
| LGGPF | 0.950385 | 0.950385 | 20.98 | 489.2587 | 5 | 6.3 | 490.26904 | 1 | 37.3777 | 740000 |
| PGAFGF | 0.967546 | 0.967546 | 12.04 | 594.2802 | 6 | 17.9 | 595.2981 | 1 | 45.3447 | 735000 |
| GPSGPPGAPGAPGAP | 0.80751 | 0.80751 | 12.33 | 1185.5778 | 15 | -17 | 593.78607 | 2 | 16.4943 | 723000 |
| PGSPGAPGLIG | 0.864111 | 0.864111 | 15.09 | 921.4919 | 11 | 1 | 461.75369 | 2 | 32.3806 | 713000 |
| GPPGPPGPGNPMGP | 0.968378 | 0.968378 | 10.26 | 1227.5706 | 14 | -11.5 | 614.78552 | 2 | 39.6731 | 710000 |
| PGGPPPPPPPP | 0.95106 | 0.95106 | 10.09 | 1005.5283 | 11 | -13.9 | 503.76447 | 2 | 36.3855 | 704000 |
| FSFLPA | 0.886488 | 0.886488 | 17.75 | 680.3533 | 6 | 0.5 | 681.36096 | 1 | 45.7444 | 678000 |
| GLWPRSPGPAP | 0.829979 | 0.829979 | 6.51 | 1133.5981 | 11 | -13 | 567.79895 | 2 | 21.8821 | 676000 |
| LPGGLL | 0.83072 | 0.83072 | 18.56 | 568.3584 | 6 | 1.7 | 569.36664 | 1 | 40.9228 | 674000 |
| LGPGLL | 0.814199 | 0.814199 | 10.02 | 568.3584 | 6 | 1.7 | 569.36664 | 1 | 40.9228 | 674000 |
| GLPGPIGPMGPA | 0.845171 | 0.845171 | 8.67 | 1062.5532 | 12 | -16.4 | 532.27515 | 2 | 31.2532 | 667000 |
| GPMGIMGPR | 0.874229 | 0.874229 | 32.91 | 914.4466 | 9 | 1 | 458.23105 | 2 | 27.7242 | 666000 |
| PGVPGPPGPPGAP | 0.84915 | 0.84915 | 15.42 | 1095.5712 | 13 | 0.9 | 548.7934 | 2 | 23.0881 | 665000 |
| PGAGGGGGGF | 0.817879 | 0.817879 | 7.29 | 732.3191 | 10 | -9.6 | 733.31927 | 1 | 45.3661 | 647000 |
| WGFP | 0.993978 | 0.993978 | 18.85 | 505.2325 | 4 | 0.4 | 506.23999 | 1 | 41.3753 | 634000 |
| FPGLA | 0.882348 | 0.882348 | 26.39 | 503.2744 | 5 | 0.1 | 504.28171 | 1 | 31.3748 | 621000 |
| FPGIA | 0.840943 | 0.840943 | 26.39 | 503.2744 | 5 | 0.1 | 504.28171 | 1 | 31.3748 | 621000 |
| LFGGL | 0.882922 | 0.882922 | 14.49 | 505.29 | 5 | 0.8 | 506.29767 | 1 | 44.524 | 620000 |
| LGGLF | 0.912553 | 0.912553 | 5.86 | 505.29 | 5 | 0.8 | 506.29767 | 1 | 44.524 | 620000 |
| LGGIF | 0.865355 | 0.865355 | 5.86 | 505.29 | 5 | 0.8 | 506.29767 | 1 | 44.524 | 620000 |
| GPSPGAMLGPSPGP | 0.845983 | 0.845983 | 5.38 | 1220.5859 | 14 | -15.1 | 611.29102 | 2 | 19.3076 | 618000 |
| PSSPGAPGL | 0.831141 | 0.831141 | 12.52 | 781.397 | 9 | 0.7 | 782.40479 | 1 | 26.6269 | 608000 |
| GPPLNLFGGS | 0.893135 | 0.893135 | 7.78 | 957.4919 | 10 | -3.8 | 479.75143 | 2 | 20.0476 | 603000 |
| GFDFSFLP | 0.941437 | 0.941437 | 19.99 | 928.433 | 8 | -0.6 | 929.43976 | 1 | 55.897 | 592000 |
| YSFGF | 0.97125 | 0.97125 | 15.41 | 619.2642 | 5 | 0.6 | 620.27185 | 1 | 47.5414 | 589000 |
| AVPFHPL | 0.854927 | 0.854927 | 17.05 | 779.433 | 7 | 0.2 | 780.44043 | 1 | 34.2284 | 583000 |
| APLPVPPPLSSPG | 0.827235 | 0.827235 | 7.94 | 1227.6863 | 13 | -11.7 | 614.84326 | 2 | 28.7303 | 558000 |
| GPSPLLGL | 0.906049 | 0.906049 | 6.23 | 752.4432 | 8 | 0.1 | 753.45056 | 1 | 40.8496 | 555000 |
| FPGYPGPK | 0.916668 | 0.916668 | 20.69 | 861.4385 | 8 | 0.6 | 431.72678 | 2 | 28.1078 | 553000 |
| LPFRFG | 0.951768 | 0.951768 | 21.29 | 735.4068 | 6 | 0.9 | 368.711 | 2 | 36.226 | 546000 |
| GVPGPPGF | 0.932283 | 0.932283 | 14.88 | 726.37 | 8 | 5.5 | 727.38129 | 1 | 37.5062 | 540000 |
| PGYGL | 0.834884 | 0.834884 | 18.16 | 505.2536 | 5 | 0.8 | 506.26132 | 1 | 23.7593 | 532000 |
| GIRAPGF | 0.838529 | 0.838529 | 5.61 | 716.3969 | 7 | -15.7 | 359.2001 | 2 | 39.5462 | 528000 |
| GFPGLPGR | 0.931118 | 0.931118 | 21.47 | 799.434 | 8 | 0.6 | 400.72455 | 2 | 26.4689 | 525000 |
| FDGPPGAP | 0.827684 | 0.827684 | 8.43 | 756.3442 | 8 | 0.7 | 757.35205 | 1 | 50.2272 | 520000 |
| GPSGPPGAPGAPGAPG | 0.833536 | 0.833536 | 12.93 | 1242.5992 | 16 | -15.1 | 622.29749 | 2 | 22.0291 | 516000 |
| GPRLL | 0.850908 | 0.850908 | 20.06 | 554.354 | 5 | 0.9 | 278.18454 | 2 | 19.6047 | 506000 |
| FSPRLP | 0.911721 | 0.911721 | 8.13 | 715.4017 | 6 | -15.8 | 716.39764 | 1 | 47.6774 | 505000 |
| PTGPAGPPGF | 0.865747 | 0.865747 | 6.33 | 896.4392 | 10 | 4 | 449.22867 | 2 | 21.9451 | 504000 |
| FLGAL | 0.846586 | 0.846586 | 14.08 | 519.3057 | 5 | 0.7 | 520.31329 | 1 | 45.7596 | 498000 |
| PGYPGAMGPP | 0.824661 | 0.824661 | 5.96 | 942.4269 | 10 | -3.6 | 472.21906 | 2 | 35.6718 | 496000 |
| FDPSILFPK | 0.927796 | 0.927796 | 30.07 | 1062.5749 | 9 | 0.7 | 532.2951 | 2 | 42.2665 | 488000 |
| GLPGPM | 0.913842 | 0.913842 | 18.36 | 570.2835 | 6 | 0.7 | 571.2912 | 1 | 25.6812 | 488000 |
| LSGFGL | 0.845337 | 0.845337 | 15.88 | 592.322 | 6 | 0.4 | 593.32953 | 1 | 40.4221 | 477000 |
| PGIPGAPGPLGN | 0.880412 | 0.880412 | 8.29 | 1045.5556 | 12 | -8.6 | 523.78058 | 2 | 47.5894 | 477000 |
| GPPGPEGPAGISGPP | 0.860869 | 0.860869 | 7.17 | 1285.6302 | 15 | 7.2 | 643.82703 | 2 | 24.9871 | 477000 |
| GHGPW | 0.939778 | 0.939778 | 17.7 | 552.2445 | 5 | -18.4 | 553.24158 | 1 | 39.6421 | 466000 |
| APGLPPRGP | 0.880145 | 0.880145 | 11.45 | 860.4868 | 9 | -12.1 | 431.24545 | 2 | 31.2184 | 464000 |
| LPGVF | 0.847656 | 0.847656 | 20.04 | 531.3057 | 5 | 0.4 | 532.31317 | 1 | 45.3661 | 463000 |
| GPSGAPGFQGLP | 0.904342 | 0.904342 | 14.27 | 1083.5349 | 12 | 1 | 542.77527 | 2 | 18.0526 | 461000 |
| PGLGYPLF | 0.944977 | 0.944977 | 12.12 | 862.4589 | 8 | 10.9 | 432.24139 | 2 | 13.8084 | 423000 |
| KPNYLWAP | 0.834383 | 0.834383 | 8.42 | 987.5178 | 8 | -15.4 | 494.75858 | 2 | 24.5788 | 421000 |
| PGAPGPPGPPGPPGAP | 0.888565 | 0.888565 | 14.49 | 1318.6669 | 16 | -3.9 | 660.3382 | 2 | 28.4995 | 420000 |
| GPSGFGF | 0.966175 | 0.966175 | 11.19 | 667.2965 | 7 | 16.7 | 668.31494 | 1 | 40.9228 | 419000 |
| GPAGPRGEPGLP | 0.832125 | 0.832125 | 15.86 | 1103.5723 | 12 | 1.4 | 552.79419 | 2 | 17.7957 | 415000 |
| GSGGGF | 0.903647 | 0.903647 | 15.15 | 480.1968 | 6 | 9.3 | 481.20859 | 1 | 46.1536 | 410000 |
| PGQPPPQPGAP | 0.810551 | 0.810551 | 11.68 | 1041.5243 | 11 | 7.8 | 521.7735 | 2 | 48.7804 | 410000 |
| SGGFDF | 0.949911 | 0.949911 | 21.36 | 628.2493 | 6 | 0.3 | 629.25671 | 1 | 31.7843 | 409000 |
| FPGFGSP | 0.884441 | 0.884441 | 22.2 | 707.3278 | 7 | -0.1 | 708.33502 | 1 | 36.0944 | 405000 |
| PGPPLPPVPPGAP | 0.909475 | 0.909475 | 7.08 | 1191.6651 | 13 | -12.4 | 596.83246 | 2 | 45.7596 | 401000 |
| GFGGF | 0.984059 | 0.984059 | 10.43 | 483.2118 | 5 | 0.6 | 484.21933 | 1 | 28.5496 | 400000 |
| PGPQGPPGPQGAP | 0.821935 | 0.821935 | 8.03 | 1155.5672 | 13 | -6.6 | 578.78705 | 2 | 41.7898 | 398000 |
| GLPPGPSPRP | 0.835715 | 0.835715 | 14.94 | 973.5345 | 10 | -12.9 | 974.52917 | 1 | 50.212 | 396000 |
| GPVSPPGNPGPAG | 0.840868 | 0.840868 | 9.29 | 1102.5407 | 13 | 0.6 | 552.27795 | 2 | 19.6809 | 391000 |
| PGIAGMFGPK | 0.865092 | 0.865092 | 5.9 | 973.5055 | 10 | 18.7 | 487.76913 | 2 | 29.9034 | 382000 |
| LGLGF | 0.852799 | 0.852799 | 5.33 | 505.29 | 5 | 1 | 506.29779 | 1 | 43.0724 | 382000 |
| LGIGF | 0.866176 | 0.866176 | 5.33 | 505.29 | 5 | 1 | 506.29779 | 1 | 43.0724 | 382000 |
| GPSGPQGPSGAPGPK | 0.861339 | 0.861339 | 13.22 | 1289.6364 | 15 | -0.6 | 645.82507 | 2 | 16.9324 | 377000 |
| GPAGPMGLTGRP | 0.859615 | 0.859615 | 11.41 | 1109.5651 | 12 | -14.2 | 555.78198 | 2 | 19.1197 | 377000 |
| PGIGFPG | 0.886475 | 0.886475 | 16.68 | 643.3329 | 7 | -0.7 | 644.33978 | 1 | 37.7122 | 373000 |
| GLGFPGP | 0.860164 | 0.860164 | 13.74 | 643.3329 | 7 | -0.7 | 644.33978 | 1 | 37.7122 | 373000 |
| GIGFPGP | 0.881893 | 0.881893 | 13.74 | 643.3329 | 7 | -0.7 | 644.33978 | 1 | 37.7122 | 373000 |
| GPPHPPA | 0.839502 | 0.839502 | 11.48 | 671.3391 | 7 | -18.2 | 672.33411 | 1 | 37.7781 | 371000 |
| GFDFSFL | 0.953803 | 0.953803 | 31.25 | 831.3803 | 7 | 0.2 | 832.3877 | 1 | 57.9625 | 367000 |
| GPPGPPGPPGPPGAP | 0.945695 | 0.945695 | 7.6 | 1247.6298 | 15 | -12.2 | 624.81458 | 2 | 25.3984 | 362000 |
| PGSPGQPLLVWPPGS | 0.842217 | 0.842217 | 5.91 | 1487.7772 | 15 | -2.5 | 744.89404 | 2 | 32.7804 | 361000 |
| FSFLPQ | 0.838255 | 0.838255 | 16.91 | 737.3748 | 6 | 0.7 | 738.38257 | 1 | 44.1444 | 354000 |
| NGQPGPMGPR | 0.813536 | 0.813536 | 5.95 | 1009.4763 | 10 | -3.7 | 505.74359 | 2 | 17.8536 | 354000 |
| EGPPGPPGPA | 0.812947 | 0.812947 | 13.15 | 874.4184 | 10 | -0.1 | 438.21646 | 2 | 26.5114 | 352000 |
| PGIFGL | 0.908643 | 0.908643 | 20.26 | 602.3428 | 6 | 1 | 603.35065 | 1 | 45.877 | 351000 |
| PGLFGL | 0.924415 | 0.924415 | 20.26 | 602.3428 | 6 | 1 | 603.35065 | 1 | 45.877 | 351000 |
| LPPWFVG | 0.950368 | 0.950368 | 20.07 | 814.4377 | 7 | -0.4 | 815.44464 | 1 | 51.5033 | 351000 |
| FGPGLL | 0.954982 | 0.954982 | 15.06 | 602.3428 | 6 | 1 | 603.35065 | 1 | 45.877 | 351000 |
| GPTGFL | 0.902122 | 0.902122 | 14.1 | 590.3064 | 6 | 0.5 | 591.31396 | 1 | 32.5954 | 346000 |
| SPPGPA | 0.800049 | 0.800049 | 13.78 | 524.2594 | 6 | 0.9 | 525.26721 | 1 | 21.783 | 345000 |
| GIPGPL | 0.893257 | 0.893257 | 15.62 | 552.3271 | 6 | 0.3 | 553.33453 | 1 | 32.9607 | 343000 |
| GLPGPL | 0.895502 | 0.895502 | 15.62 | 552.3271 | 6 | 0.3 | 553.33453 | 1 | 32.9607 | 343000 |
| PGRPVWPPGPA | 0.882285 | 0.882285 | 5.87 | 1129.6032 | 11 | 9.6 | 565.81433 | 2 | 33.9334 | 343000 |
| GFPGLNGLNG | 0.810846 | 0.810846 | 10.62 | 944.4715 | 10 | 1.6 | 473.2438 | 2 | 26.1239 | 329000 |
| FGVSPGF | 0.862375 | 0.862375 | 19.49 | 709.3435 | 7 | -0.3 | 710.35059 | 1 | 42.5128 | 327000 |
| PPMPGPPPLG | 0.947074 | 0.947074 | 5.81 | 958.4946 | 10 | -19.5 | 480.24521 | 2 | 37.8078 | 326000 |
| FYFG | 0.984344 | 0.984344 | 10.2 | 532.2322 | 4 | 1.3 | 533.24011 | 1 | 48.7561 | 324000 |
| SGPPGGFGPLGPQGLPGPSG | 0.936808 | 0.936808 | 6.43 | 1731.858 | 20 | 13.5 | 866.94794 | 2 | 40.9378 | 324000 |
| PGSFLGGPA | 0.857326 | 0.857326 | 7.35 | 801.4021 | 9 | 0 | 802.40936 | 1 | 44.521 | 323000 |
| PAGPVSPPGNPGPAG | 0.834785 | 0.834785 | 5.8 | 1270.6305 | 15 | -7.5 | 636.31781 | 2 | 37.3449 | 322000 |
| LYFFPSLR | 0.901991 | 0.901991 | 10.86 | 1041.5647 | 8 | -13.5 | 521.78259 | 2 | 42.4617 | 317000 |
| IPGFPGA | 0.806909 | 0.806909 | 18.81 | 657.3486 | 7 | 1.5 | 658.35681 | 1 | 41.4463 | 315000 |
| LPGFPGA | 0.82788 | 0.82788 | 18.81 | 657.3486 | 7 | 1.5 | 658.35681 | 1 | 41.4463 | 315000 |
| GPPGPPGPPGAP | 0.926113 | 0.926113 | 7.45 | 996.5028 | 12 | -8.9 | 499.25424 | 2 | 48.7604 | 312000 |
| NFYADFG | 0.8692 | 0.8692 | 7.06 | 832.3391 | 7 | -0.1 | 833.34631 | 1 | 45.0045 | 312000 |
| SPGPILGPSPGPGPSP | 0.842648 | 0.842648 | 19.89 | 1412.7299 | 16 | 7.8 | 707.37775 | 2 | 32.6611 | 308000 |
| GPGPGPGPAAAPGPS | 0.861388 | 0.861388 | 17.6 | 1185.5778 | 15 | -15.8 | 593.7868 | 2 | 30.7435 | 304000 |
| PGDAGFPGLPGS | 0.806159 | 0.806159 | 11.22 | 1070.5032 | 12 | 0.3 | 536.25903 | 2 | 28.3279 | 301000 |
| PSGGFDF | 0.937625 | 0.937625 | 25.66 | 725.302 | 7 | -0.1 | 726.3092 | 1 | 35.2243 | 291000 |
| PGSMGLPGKHGLPG | 0.838786 | 0.838786 | 7.5 | 1303.6706 | 14 | 6.3 | 652.84668 | 2 | 35.8347 | 279000 |
| GIPGMP | 0.894067 | 0.894067 | 19 | 570.2835 | 6 | -0.2 | 571.29071 | 1 | 22.0761 | 273000 |
| GLPGMP | 0.911703 | 0.911703 | 19 | 570.2835 | 6 | -0.2 | 571.29071 | 1 | 22.0761 | 273000 |
| PSGPQGPSGAPGPK | 0.820322 | 0.820322 | 11.49 | 1232.6149 | 14 | 0.1 | 617.31482 | 2 | 20.0793 | 273000 |
| PGHPFIM | 0.945631 | 0.945631 | 18.86 | 797.3894 | 7 | 0.3 | 399.70209 | 2 | 38.2944 | 270000 |
| GAPPPHPYPPQ | 0.8621 | 0.8621 | 11.68 | 1156.5665 | 11 | -8.3 | 579.28571 | 2 | 48.2594 | 269000 |
| PGLPGFPGPEGP | 0.853573 | 0.853573 | 15.54 | 1120.5553 | 12 | 19.2 | 561.29565 | 2 | 39.5793 | 268000 |
| PGFHP | 0.893588 | 0.893588 | 18.66 | 553.2649 | 5 | 0.1 | 554.27222 | 1 | 25.3722 | 264000 |
| GLWPRSPGPA | 0.801314 | 0.801314 | 6.61 | 1036.5454 | 10 | 10.7 | 519.28552 | 2 | 32.5677 | 264000 |
| GPAKLFGFVA | 0.840029 | 0.840029 | 5.72 | 1005.5647 | 10 | -3.8 | 503.78772 | 2 | 37.4438 | 263000 |
| QPPPPAQPPAPGGGPQ | 0.84856 | 0.84856 | 11.99 | 1491.747 | 16 | 10 | 746.88824 | 2 | 49.9114 | 262000 |
| FGFGPS | 0.831117 | 0.831117 | 11.31 | 610.2751 | 6 | 0.6 | 611.28271 | 1 | 32.5954 | 258000 |
| RYPW | 0.937492 | 0.937492 | 14.17 | 620.3071 | 4 | -16.8 | 621.30389 | 1 | 36.9531 | 256000 |
| GLLAF | 0.846921 | 0.846921 | 13.23 | 519.3057 | 5 | 0.2 | 520.31305 | 1 | 41.0913 | 256000 |
| GLFPL | 0.951328 | 0.951328 | 16.77 | 545.3213 | 5 | 0.7 | 546.32898 | 1 | 46.072 | 252000 |
| GLFPI | 0.879364 | 0.879364 | 16.77 | 545.3213 | 5 | 0.7 | 546.32898 | 1 | 46.072 | 252000 |
| PGPPGPPGPA | 0.928344 | 0.928344 | 5.52 | 842.4286 | 10 | 0.2 | 843.43604 | 1 | 49.872 | 251000 |
| GIGFSGAPGPPGPA | 0.812121 | 0.812121 | 13.39 | 1180.5876 | 14 | -11.4 | 591.29437 | 2 | 13.3678 | 249000 |
| GMPGGLL | 0.891212 | 0.891212 | 17.3 | 643.3363 | 7 | -5.3 | 644.34015 | 1 | 40.6446 | 248000 |
| GPAPGPPPA | 0.886978 | 0.886978 | 24.74 | 759.3915 | 9 | 0.7 | 380.70328 | 2 | 20.5388 | 246000 |
| WPGF | 0.995408 | 0.995408 | 11.6 | 505.2325 | 4 | 1 | 506.24026 | 1 | 41.7931 | 245000 |
| DPPGL | 0.868012 | 0.868012 | 16.34 | 497.2485 | 5 | 1 | 498.25632 | 1 | 47.9069 | 244000 |
| FDFSFLPQ | 0.836422 | 0.836422 | 14.56 | 999.4702 | 8 | -0.5 | 1000.47693 | 1 | 54.5651 | 239000 |
| GPSIFGL | 0.908273 | 0.908273 | 6.78 | 689.3748 | 7 | 0.5 | 690.38239 | 1 | 46.1536 | 237000 |
| GPPGPPGFPGPTGQP | 0.917149 | 0.917149 | 5.24 | 1358.6618 | 15 | -1.8 | 453.89374 | 3 | 16.1619 | 233000 |
| PGFF | 0.994302 | 0.994302 | 16.7 | 466.2216 | 4 | 8.7 | 467.23291 | 1 | 35.5801 | 228000 |
| QPGDFGESGFPGI | 0.827548 | 0.827548 | 5.28 | 1306.5829 | 13 | 10.8 | 654.30579 | 2 | 20.2802 | 228000 |
| PGPPGPPGPPGAP | 0.932933 | 0.932933 | 12.73 | 1093.5556 | 13 | -3.7 | 547.78302 | 2 | 18.6433 | 227000 |
| GHPGPPGPPGEQGLP | 0.858574 | 0.858574 | 5.48 | 1392.6786 | 15 | 8.9 | 697.35272 | 2 | 37.2019 | 224000 |
| GPGTPGPPVRMGPA | 0.835897 | 0.835897 | 7.19 | 1289.655 | 14 | 16 | 645.84509 | 2 | 20.5111 | 222000 |
| NGSPGPMGP | 0.844927 | 0.844927 | 10.63 | 812.3487 | 9 | -18.8 | 813.34064 | 1 | 31.9098 | 221000 |
| PGPPVRMGPAPG | 0.834931 | 0.834931 | 6.85 | 1131.5859 | 12 | 14.7 | 566.80853 | 2 | 35.6446 | 221000 |
| IGFPGR | 0.859653 | 0.859653 | 12.64 | 645.3598 | 6 | 0.3 | 323.68729 | 2 | 20.8533 | 218000 |
| GPPGLPGAP | 0.871909 | 0.871909 | 28.13 | 761.4071 | 9 | 0.7 | 381.71112 | 2 | 24.4795 | 217000 |
| GLSDPFARVFFIT | 0.857387 | 0.857387 | 11.5 | 1468.7714 | 13 | -2.7 | 735.39099 | 2 | 36.7346 | 216000 |
| GRPPGSPF | 0.90097 | 0.90097 | 10.53 | 813.4133 | 8 | 1.3 | 407.71448 | 2 | 32.1032 | 214000 |
| GPSGFPGLAG | 0.903956 | 0.903956 | 16.83 | 858.4235 | 10 | 1.7 | 430.21979 | 2 | 13.8168 | 211000 |
| MGRPGFP | 0.906608 | 0.906608 | 11.87 | 760.369 | 7 | 8 | 761.38239 | 1 | 38.7102 | 208000 |
| GPGLPGMP | 0.931634 | 0.931634 | 19.09 | 724.3578 | 8 | 1.3 | 725.36597 | 1 | 27.0068 | 201000 |
| LGPPLLG | 0.828203 | 0.828203 | 8.52 | 665.4112 | 7 | 0.6 | 666.41882 | 1 | 43.8158 | 200000 |
| FLSPG | 0.827536 | 0.827536 | 11.14 | 519.2693 | 5 | -0.2 | 520.27643 | 1 | 26.6867 | 199000 |
| PSPPRNL | 0.808534 | 0.808534 | 8.33 | 779.429 | 7 | 0.7 | 390.72205 | 2 | 30.1936 | 198000 |
| GPPGLPGYQGAP | 0.824775 | 0.824775 | 5.2 | 1109.5505 | 12 | -9.8 | 555.7771 | 2 | 11.066 | 196000 |
| PPGSPP | 0.81379 | 0.81379 | 16.46 | 550.2751 | 6 | 7.2 | 551.28632 | 1 | 22.0761 | 195000 |
| IPGWASLFT | 0.812603 | 0.812603 | 9.32 | 990.5174 | 9 | -14.4 | 496.25888 | 2 | 53.3028 | 195000 |
| PGYPFGLKGR | 0.843841 | 0.843841 | 7.82 | 1090.5923 | 10 | -19.8 | 546.29266 | 2 | 29.0254 | 191000 |
| GPGRGP | 0.819679 | 0.819679 | 15.31 | 539.2816 | 6 | 4.1 | 540.29108 | 1 | 37.8766 | 189000 |
| GPGPSPGPILGPSPGP | 0.90116 | 0.90116 | 15.21 | 1382.7194 | 16 | 0.2 | 692.36713 | 2 | 42.4638 | 189000 |
| GLFGF | 0.976866 | 0.976866 | 12.8 | 539.2744 | 5 | 17.5 | 540.29108 | 1 | 37.8766 | 189000 |
| GIFGF | 0.967021 | 0.967021 | 12.8 | 539.2744 | 5 | 17.5 | 540.29108 | 1 | 37.8766 | 189000 |
| PGSPGPLGR | 0.90729 | 0.90729 | 9.05 | 836.4504 | 9 | 1.8 | 419.23325 | 2 | 13.8905 | 189000 |
| PGPQGYPGL | 0.890272 | 0.890272 | 12.97 | 884.4392 | 9 | -2.8 | 443.22565 | 2 | 13.5635 | 187000 |
| GPSPGAMLGPSPG | 0.857488 | 0.857488 | 13.75 | 1123.5332 | 13 | 16.7 | 562.78326 | 2 | 30.1774 | 186000 |
| AVGGIGPPGF | 0.807606 | 0.807606 | 15.33 | 870.4599 | 10 | -3.1 | 436.2359 | 2 | 19.7968 | 184000 |
| GPPGPPGPRGPPGPA | 0.949952 | 0.949952 | 11.61 | 1306.6782 | 15 | -18.7 | 654.33417 | 2 | 44.1822 | 184000 |
| PGTPGGIGFSGAPGPPGPAG | 0.87166 | 0.87166 | 6.28 | 1646.8052 | 20 | -9.4 | 549.93719 | 3 | 20.0476 | 177000 |
| GPALPPQGL | 0.872534 | 0.872534 | 14.13 | 848.4756 | 9 | 0.2 | 425.24515 | 2 | 32.8337 | 173000 |
| GPSGVPPGMP | 0.863646 | 0.863646 | 11.6 | 894.4269 | 10 | 0.6 | 448.22101 | 2 | 27.7874 | 173000 |
| PGPSGPPGLAGP | 0.916851 | 0.916851 | 11.05 | 1002.5134 | 12 | 1.3 | 502.26462 | 2 | 23.5959 | 173000 |
| PGFPGAPG | 0.865803 | 0.865803 | 6.57 | 698.3387 | 8 | -15.3 | 699.33533 | 1 | 45.3438 | 172000 |
| PGPPGPPGSP | 0.856983 | 0.856983 | 10.89 | 858.4235 | 10 | 2.1 | 859.43262 | 1 | 43.2021 | 170000 |
| GSPGFGLG | 0.922804 | 0.922804 | 10.78 | 690.3337 | 8 | 1.7 | 691.3421 | 1 | 29.189 | 170000 |
| GLPGYPGR | 0.846999 | 0.846999 | 7.52 | 815.429 | 8 | 1.4 | 408.72232 | 2 | 21.6647 | 167000 |
| GGFDFSFLPQPP | 0.847815 | 0.847815 | 6.01 | 1307.6186 | 12 | 0.7 | 654.81702 | 2 | 53.9695 | 166000 |
| PEGKPGMPGF | 0.865751 | 0.865751 | 5.73 | 1015.4797 | 10 | -16.8 | 508.73856 | 2 | 32.8987 | 166000 |
| PNYLWAP | 0.865957 | 0.865957 | 6.85 | 859.4228 | 7 | -18.4 | 860.41431 | 1 | 60.0913 | 165000 |
| FDFSGLRLDW | 0.815433 | 0.815433 | 9.13 | 1254.6033 | 10 | 0.8 | 628.30945 | 2 | 44.2141 | 163000 |
| QGPPGPSGFTGQPGA | 0.817702 | 0.817702 | 6.93 | 1353.6313 | 15 | 19 | 677.83575 | 2 | 34.5537 | 163000 |
| GPMGPR | 0.891703 | 0.891703 | 35.28 | 613.3006 | 6 | 1.7 | 307.65811 | 2 | 8.9677 | 157000 |
| GERGFPGFPG | 0.845503 | 0.845503 | 11.49 | 1019.4824 | 10 | -2 | 510.7475 | 2 | 8.8631 | 157000 |
| FSGLGL | 0.867165 | 0.867165 | 16.54 | 592.322 | 6 | -0.3 | 593.32916 | 1 | 42.9562 | 156000 |
| GAMGPPGL | 0.901719 | 0.901719 | 6.22 | 698.3421 | 8 | -5.1 | 350.17654 | 2 | 25.3722 | 156000 |
| PGMPPQP | 0.839897 | 0.839897 | 22.1 | 722.3421 | 7 | -3.3 | 723.34705 | 1 | 36.8226 | 154000 |
| PGSGPPGGPGRK | 0.909944 | 0.909944 | 9.68 | 1062.557 | 12 | 13.5 | 532.29297 | 2 | 33.0507 | 154000 |
| APLGDFFGGELGPA | 0.807849 | 0.807849 | 5.01 | 1346.6506 | 14 | 3.6 | 674.33502 | 2 | 46.0503 | 154000 |
| GPMGPAGPR | 0.860452 | 0.860452 | 30.62 | 838.4119 | 9 | 2.5 | 420.21429 | 2 | 14.1335 | 153000 |
| GPAGPRFG | 0.939843 | 0.939843 | 17.75 | 757.3871 | 8 | 2.3 | 379.70169 | 2 | 16.6458 | 151000 |
| PGPPGAPSGGFDFSFLPQP | 0.943495 | 0.943495 | 5.31 | 1870.8889 | 19 | 0 | 936.45178 | 2 | 53.0428 | 150000 |
| GPAGLRGF | 0.901402 | 0.901402 | 22.78 | 773.4184 | 8 | -14.4 | 387.71091 | 2 | 38.8896 | 148000 |
| GFDFGL | 0.965112 | 0.965112 | 20.84 | 654.3013 | 6 | 1 | 655.3092 | 1 | 48.079 | 148000 |
| FGLGGL | 0.83813 | 0.83813 | 16.32 | 562.3115 | 6 | 0.7 | 563.31915 | 1 | 47.9677 | 147000 |
| FGLGLG | 0.823367 | 0.823367 | 14.26 | 562.3115 | 6 | 0.7 | 563.31915 | 1 | 47.9677 | 147000 |
| AGPPGPPGFPGPTGQP | 0.902223 | 0.902223 | 5.88 | 1429.699 | 16 | -12.5 | 715.84784 | 2 | 37.7573 | 147000 |
| FSFLPQPP | 0.881369 | 0.881369 | 12.43 | 931.4803 | 8 | 1.6 | 466.7482 | 2 | 48.4983 | 146000 |
| PGPIGWPGPMGPK | 0.962933 | 0.962933 | 11.4 | 1289.659 | 13 | -15.3 | 430.88702 | 3 | 15.6848 | 142000 |
| GILRGF | 0.817202 | 0.817202 | 7.87 | 661.3911 | 6 | 0.1 | 331.70288 | 2 | 31.4339 | 139000 |
| LGPPGYPGPA | 0.823201 | 0.823201 | 7.44 | 924.4705 | 10 | -0.4 | 463.24234 | 2 | 32.9281 | 137000 |
| WGGPG | 0.956861 | 0.956861 | 14.34 | 472.207 | 5 | 7.8 | 473.21796 | 1 | 60.83 | 136000 |
| DLPLGLM | 0.809207 | 0.809207 | 15.88 | 757.4044 | 7 | -4.2 | 758.40845 | 1 | 49.5116 | 135000 |
| GPPGPPGEQGLPGPS | 0.868962 | 0.868962 | 11.74 | 1342.6517 | 15 | 4 | 672.33582 | 2 | 23.5327 | 134000 |
| PGHPGLP | 0.845033 | 0.845033 | 21.66 | 673.3547 | 7 | 0.4 | 337.68478 | 2 | 15.8113 | 132000 |
| GPPGYPGKP | 0.903655 | 0.903655 | 14.53 | 868.4443 | 9 | -0.1 | 435.22937 | 2 | 18.0298 | 131000 |
| FHPIHPP | 0.804756 | 0.804756 | 10.66 | 843.4391 | 7 | 1 | 282.15393 | 3 | 20.0793 | 131000 |
